# Kynureninase Is a Prognostic Biomarker of Poor Survival and Gemcitabine Resistance in Pancreatic Ductal Adenocarcinoma

**DOI:** 10.3390/ijms27219820

**Published:** 2026-11-01

**Authors:** Baris Mercanoglu, Nina Schraps, Anastasios D. Giannou, Siwen Zhang, Eleftherios D. Papazoglou, Cornelia Schroeder, Ziyang Hong, Marcel Kwiatkowski, Hartmut Schlüter, Faik Güntac Uzunoglu, Rainer Grotelüschen, Nathaniel Melling, Maximilian Bockhorn, Thilo Hackert, Gerrit Wolters-Eisfeld, Cenap Güngör

**Affiliations:** 1 Department of General, Visceral, Transplant and Thoracic Surgery, University Medical Center Hamburg-Eppendorf, 20246 Hamburg, Germany; n.schraps@uke.de (N.S.); ge.wolters@uke.de (G.W.-E.); 2 Institute of Pathology, University Medical Center Hamburg-Eppendorf, 20246 Hamburg, Germany; 3 Section of Molecular Immunology und Gastroenterology, I. Department of Medicine, University Medical Center Hamburg-Eppendorf, 20246 Hamburg, Germany; 4 Department of Surgery, Division of General Surgery, University of British Columbia, Vancouver, BC V5Z 1M9, Canada; 5 Department of Urologic Sciences, University of British Columbia, Vancouver, BC V5Z 1M9, Canada; 6 Department of Biomedicine, University of Bergen, 5020 Bergen, Norway; 7 Mass Spectrometric Proteomics—Institute for Clinical Chemistry & Laboratory Medicine, University Medical Center Hamburg-Eppendorf, 20246 Hamburg, Germany; 8 Department of General and Visceral Surgery, University Medical Center Oldenburg, 26133 Oldenburg, Germany

**Keywords:** pancreatic cancer, PDAC, kynureninase, KYNU, tryptophan metabolism, cancer metabolism, chemoresistance, gemcitabine resistance

## Abstract

Pancreatic ductal adenocarcinoma (PDAC) remains one of the deadliest malignancies, largely due to intrinsic or acquired resistance to systemic therapy. In addition to dysregulated glucose metabolism, emerging evidence implicates amino acid metabolism as a key determinant of tumor growth and treatment adaptation. To identify biomarkers associated with chemoresistance, we performed SILAC-based mass spectrometry secretome profiling of gemcitabine-resistant and chemosensitive PDAC cell models, followed by qRT-PCR and immunoblot validation. Functional relevance was assessed by stable shRNA-mediated KYNU knockdown, proliferation assays, and Transwell migration assays. Clinical significance was evaluated by immunohistochemical analysis of KYNU expression in a PDAC tissue microarray (*n* = 368) and correlation with overall survival. Transcriptomic and pathway enrichment analyses were used to characterize resistance-associated programs. Kynureninase (KYNU), a tryptophan-catabolic enzyme, was upregulated and preferentially secreted by gemcitabine-resistant cells and induced by gemcitabine, interferon-γ, and hypoxia. KYNU knockdown significantly reduced proliferation without affecting migration. Resistant cells displayed higher KYNU expression and a broader therapy-adapted cellular state. In patient samples, high KYNU expression was associated with significantly shorter overall survival (*p* = 0.0198). These findings identify KYNU as a marker of a therapy-adapted PDAC phenotype and support its potential as a prognostic and treatment-response biomarker.

## 1. Introduction

With a median overall survival of 4 months and a 5-year survival rate of approximately 13%, pancreatic cancer is one of the most common causes of cancer-related death worldwide [1]. The high mortality is substantially driven by nonspecific symptoms and a frequent presence of metastases at diagnosis [1,2,3,4]. In addition, an aggressive tumor biology contributes to an increased resistance to chemo- and immunotherapies [1,5,6,7]. Gemcitabine has long been considered the gold standard in the treatment course of advanced pancreatic cancer [8,9]. Due to limited efficacy and frequent intrinsic as well as acquired chemoresistance to gemcitabine monotherapy, the current therapy regimen for metastatic pancreatic cancer includes FOLFIRINOX (5-fluorouracil, leucovorin, irinotecan and oxaliplatin) and the combination of gemcitabine and nab-paclitaxel [1,10,11,12,13,14]. However, chemotherapy resistance remains a key problem in the treatment landscape of pancreatic cancer [8,15]. A deeper understanding of the molecular drivers of pancreatic cancer and its resistance mechanisms to common chemotherapies is urgently needed in order to improve patient outcomes.

In addition to glucose metabolism playing a central role in tumor cell energy production, amino acid metabolism substantially supports cancer cell growth [16,17]. The metabolic plasticity of cancer cells enables them to use amino acids from both endogenous synthesis and exogenous sources to maintain rapid proliferation and adapt to nutrient-poor microenvironments [18,19,20,21]. Kynureninase (KYNU) is a pyridoxal-5′-phosphate-dependent enzyme and a member of the aminotransferase superfamily that plays a central role in tryptophan catabolism and the synthesis of NAD cofactors [22,23]. In eukaryotes, the enzyme catalyzes the hydrolytic cleavage of 3-hydroxy-L-kynurenine to 3-hydroxyanthranilic acid and L-alanine [23]. In cancer, KYNU expression is associated with an immunosuppressive tumor microenvironment, increased expression of immune checkpoint genes, and increased infiltration of regulatory T-cells [24,25]. Upregulation of KYNU expression has been found in several cancer types such as prostate, colon, gastric, renal cell, and lung cancer and has been linked to poor patient outcomes in some of these tumor entities [24,25,26,27].

The aim of this study was to identify new biomarkers for chemoresistant pancreatic cancer. By examining the secretome of chemoresistant pancreatic ductal adenocarcinoma (PDAC) cells, we identified an overexpressed and secreted form of the KYNU protein in chemoresistant PDAC cells. Based on these findings, we investigated whether KYNU expression is altered in PDAC cell lines upon chemotherapy exposure and whether short hairpin RNA (shRNA)-mediated KYNU depletion affects cancer cell proliferation and migration in vitro.

## 2. Results

### 2.1. KYNU Is Overexpressed in PDAC and Displays Nuclear and Cytoplasmic Localization in Chemoresistant Cells

**Figure 1:**
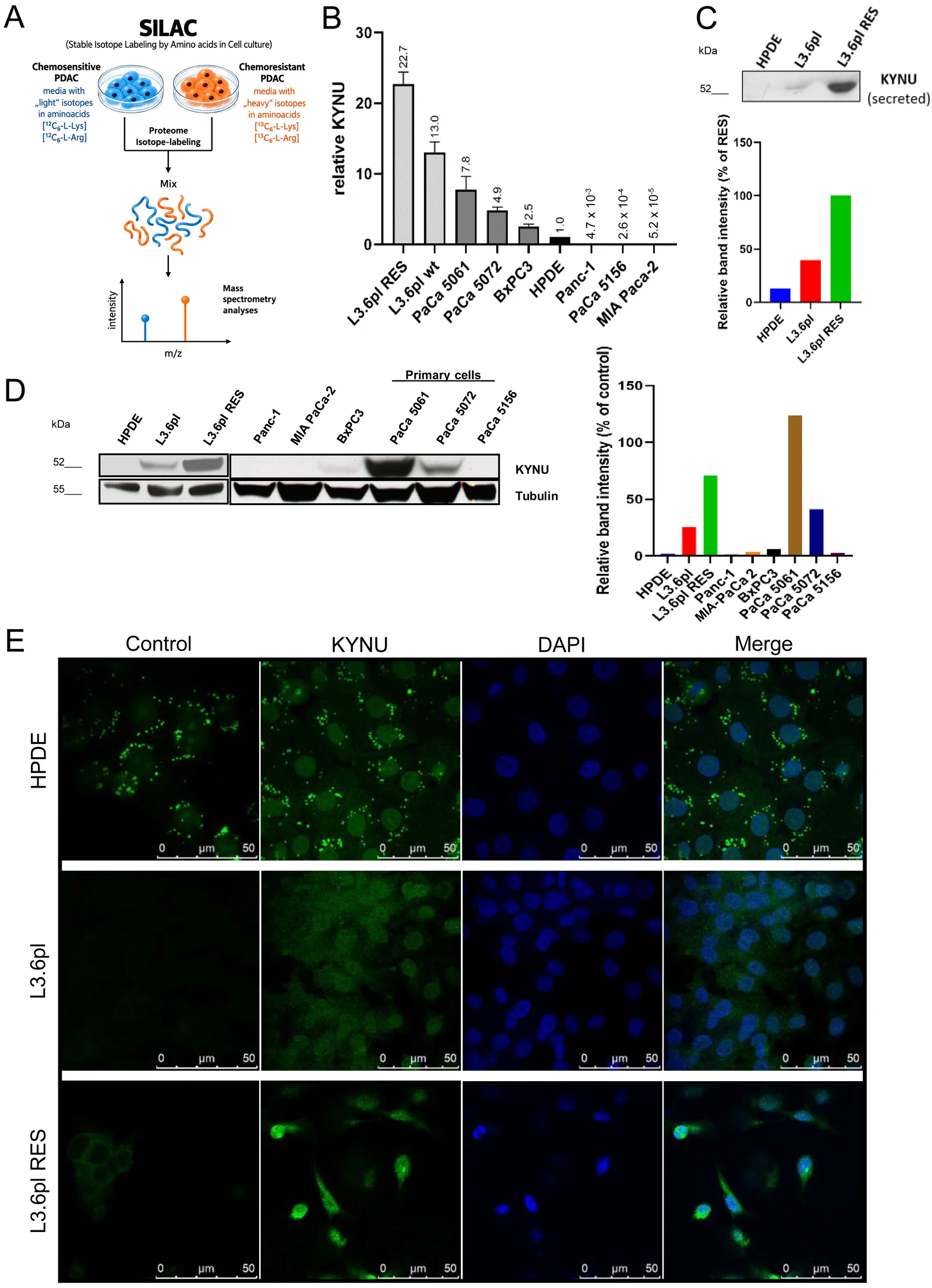
**Differential KYNU expression, secretion, and subcellular localization in pancreatic cancer cell models.** (**A**) Schematic overview of the SILAC-based secretome survey involving the incorporation of stable isotopes to compare total cellular proteomes. (**B**) KYNU mRNA expression in PDAC cell lines relative to the nonmalignant human pancreatic ductal epithelial cell line HPDE, as determined by qRT-PCR. Expression levels are presented as fold change relative to HPDE. (**C**) KYNU secretion was assessed by Western blotting of cell-free and concentrated culture supernatants from L3.6pl and L3.6pl RES cells. Increased KYNU secretion was detected in L3.6pl RES; it was not detectable in HPDE supernatants. The intensity of protein bands was quantified using Fiji software(ImageJ version 2.16.0/1.54p). (**D**) KYNU protein levels in cell lysates from PDAC cell lines and HPDE were assessed by Western blotting; α-tubulin served as a loading control. KYNU expression was increased in gemcitabine-resistant L3.6pl RES cells, compared with the chemosensitive parental L3.6pl line. Band intensities were quantified by densitometric analysis using Fiji. (**E**) Immunofluorescence staining of endogenous KYNU in HPDE, L3.6pl, and L3.6pl RES cells using an anti-KYNU primary antibody and a fluorescein-conjugated secondary antibody (green); nuclei were counterstained with DAPI (blue). KYNU displayed cytoplasmic and nuclear localization in L3.6pl RES cells. Cells were grown on coverslips and fixed with paraformaldehyde. Images were acquired at 400× magnification.

To identify biomarkers associated with chemoresistance in PDAC, we used a SILAC-based discovery strategy for the simultaneous identification and relative quantification of proteins in chemoresistant versus chemosensitive PDAC cell models. Secretome profiling revealed kynureninase as a candidate protein enriched in the conditioned culture medium of gemcitabine-resistant L3.6pl RES cells compared with the parental, gemcitabine-sensitive L3.6pl line (Figure 1A).

Because the expression pattern and potential relevance of KYNU in PDAC had not been examined previously, we next assessed KYNU expression across a panel of pancreatic cell models. qRT-PCR analyses revealed elevated KYNU mRNA levels in multiple PDAC cell lines, including L3.6pl, L3.6pl RES, and BxPC-3, as well as in primary PDAC cell lines (PaCa 5061 and PaCa 5072), whereas KYNU transcripts were undetectable in nonmalignant HPDE cells. Notably, KYNU mRNA expression was significantly higher in L3.6pl RES cells than in parental L3.6pl cells (Figure 1B). Consistent with these transcript data, immunoblotting confirmed robust intracellular KYNU protein expression in PDAC cells, with increased KYNU protein abundance in L3.6pl RES cells relative to L3.6pl (Figure 1D). In line with the SILAC secretome findings, Western blot analysis of cell-free culture supernatants detected higher levels of secreted KYNU in L3.6pl RES compared with L3.6pl, while KYNU was undetectable in HPDE supernatants (Figure 1C). Together, these data indicate that KYNU is upregulated in PDAC cell models and is preferentially secreted by chemoresistant cells.

Given that metabolic enzymes such as KYNU have been reported to localize predominantly to the cytoplasm in hepatocytes [28], we performed subcellular localization studies to further characterize KYNU distribution in PDAC. Immunofluorescence staining revealed a cytoplasmic and nuclear KYNU signal in PDAC cells (Figure 1E). In HPDE cells, interpretation was limited by strong autofluorescence, precluding reliable assessment of KYNU-specific signal. To corroborate these observations biochemically, we performed subcellular fractionation of L3.6pl and L3.6pl RES cells, which confirmed the presence of KYNU in both cytoplasmic and nuclear fractions. Notably, the nuclear fraction contained a KYNU-immunoreactive band migrating at approximately 100 kDa (Supplementary Figure S1), consistent with a potentially higher-molecular-weight KYNU species. As KYNU has been previously reported to form homodimers in purified preparations [29], these preliminary data may be pointing to the presence of a presumably dimeric KYNU form in the nuclear compartment of PDAC cells. Because the higher-molecular-weight KYNU band may also reflect post-translational modification, the molecular identity of this species requires further experimental validation in future studies.

### 2.2. KYNU Knockdown Reduces Proliferation and Enhances Gemcitabine Sensitivity in Resistant PDAC Cells

**Figure 2:**
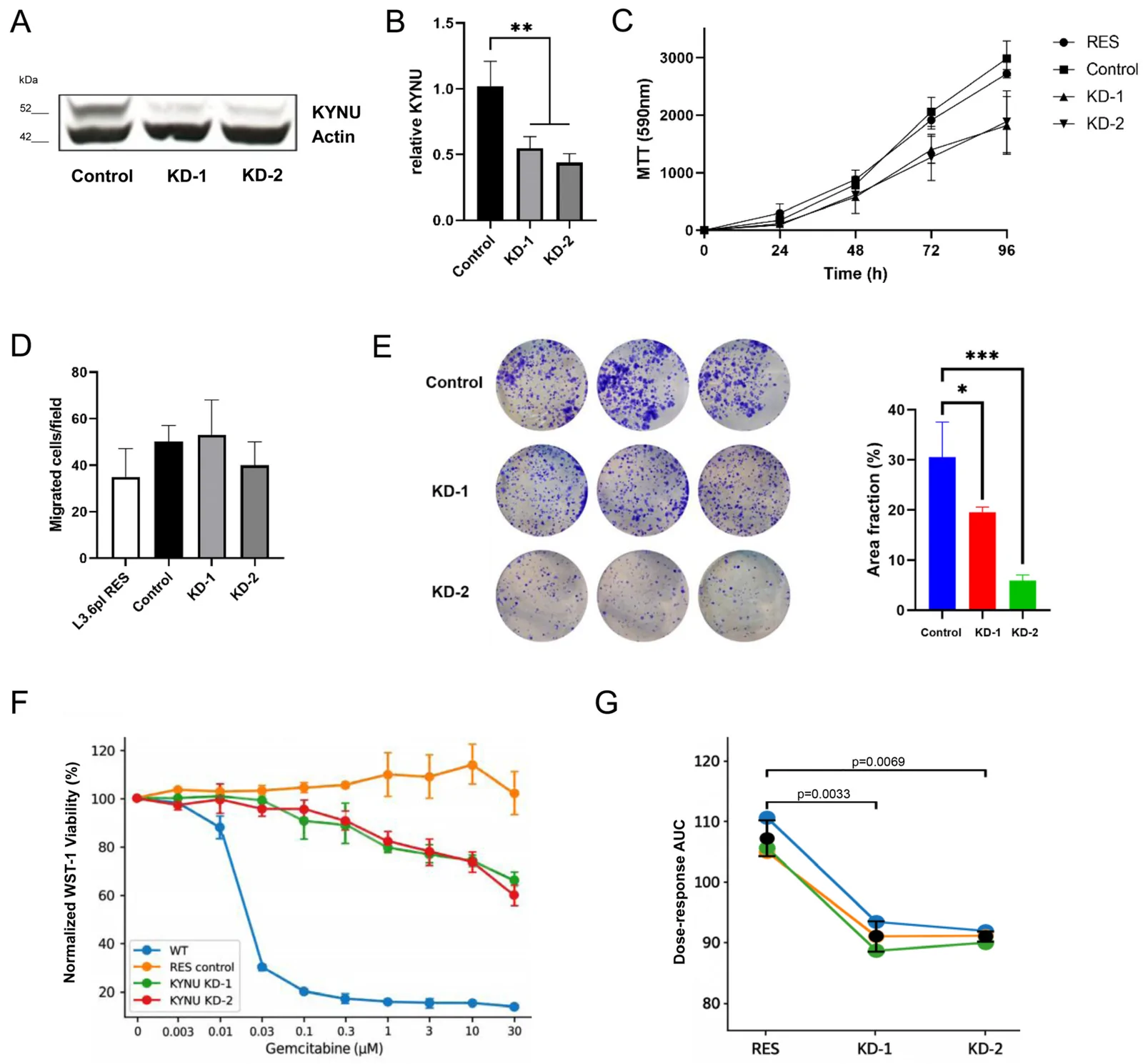
**KYNU knockdown reduces proliferation and colony formation and enhances gemcitabine sensitivity in resistant PDAC cells.** Stable KYNU knockdown was established in gemcitabine-resistant L3.6pl cells using γ-retroviral vectors encoding two independent KYNU-targeting shRNAs (KD-1 and KD-2). Non-targeting shRNA-transduced cells served as controls (sh-Control). (**A**,**B**) Knockdown efficiency was confirmed by Western blotting (**A**), with β-actin as a loading control, and qRT-PCR (B; ** *p* < 0.01). (**C**) Cell proliferation was assessed by MTT assay over 96 h. KYNU knockdown reduced proliferation, reaching statistical significance for KD-1 at 72 h (*p* = 0.030) and 96 h (*p* = 0.036). (**D**) Transwell migration assays revealed no significant differences between control and knockdown cells. (**E**) Representative crystal violet-stained colonies and quantification of colony-covered area following 14 days of gemcitabine treatment. KYNU knockdown significantly reduced colony formation (mean ± SD, three replicate wells; * *p* < 0.05, *** *p* < 0.001). (**F**) Gemcitabine dose–response curves of parental sensitive L3.6pl cells (WT), resistant sh-Control cells, and KYNU-knockdown derivatives following 48 h of treatment (0–30 µM). WST-1 measurements were normalized to each population’s untreated control (100% viability). Data represent mean ± SD from three independent experiments performed in technical duplicates. (**G**) Gemcitabine sensitivity was quantified by area under the dose–response curve (AUC) analysis over 0.003–30 µM using log_10_-transformed concentrations and viability values normalized to the respective untreated controls. Lines connect matched independent experiments. KYNU knockdown significantly reduced AUC compared with resistant sh-Control cells (KD-1, *p* = 0.0033; KD-2, *p* = 0.0069; paired two-tailed *t*-tests), indicating partial restoration of gemcitabine sensitivity.

To investigate the functional role of KYNU in gemcitabine resistance, stable KYNU-knockdown populations were generated in L3.6pl RES cells using two independent shRNA constructs (KD-1 and KD-2). Successful knockdown was confirmed by Western blotting (Figure 2A) and qRT-PCR (Figure 2B; *p* < 0.01). KYNU depletion reduced cell proliferation, reaching statistical significance for KD-1 at 72 h (*p* = 0.030) and 96 h (*p* = 0.036), while KD-2 showed a similar trend (Figure 2C). In contrast, Transwell assays revealed no significant changes in cell migration (Figure 2D). KYNU knockdown also significantly reduced colony formation following prolonged gemcitabine exposure (Figure 2E). To assess the contribution of KYNU to chemoresistance, gemcitabine dose–response experiments were performed in parental sensitive L3.6pl cells, resistant controls, and KYNU-knockdown populations. KYNU depletion increased gemcitabine sensitivity, as demonstrated by reduced viability following 48 h of treatment (Figure 2F). AUC analysis of dose–response curves normalized to each population’s untreated control confirmed significantly reduced AUC values in both knockdown populations compared with resistant controls (KD-1, *p* = 0.0033; KD-2, *p* = 0.0069; Figure 2G). However, sensitivity remained below that of parental cells.

Together, these findings suggest that KYNU supports proliferation and contributes to gemcitabine resistance in PDAC cells, whereas its depletion does not significantly affect migration.

### 2.3. KYNU Expression Is Upregulated in PDAC Cell Lines upon Gemcitabine Treatment

To further delineate the relationship between chemotherapy exposure and KYNU expression, we treated a panel of PDAC cell models with gemcitabine for 24 h and subsequently assessed KYNU expression. To capture potential induction across different baseline expression levels, we selected cell lines with low (Panc-1), intermediate (BxPC-3), and high (primary PaCa 5061) KYNU expression.

**Figure 3:**
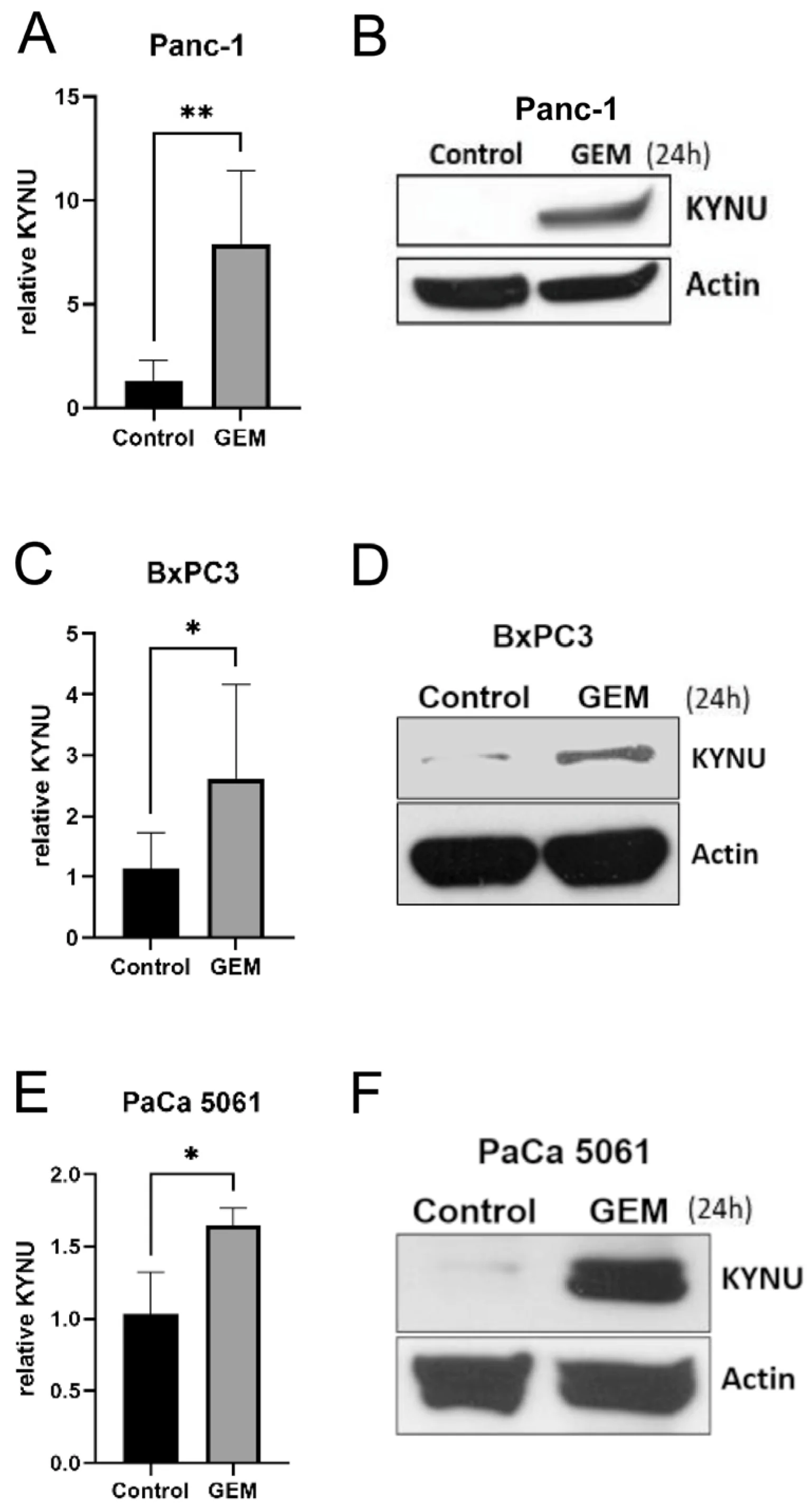
**Gemcitabine induces KYNU mRNA and protein expression in PDAC cell lines.** Panc-1, BxPC-3, and primary patient-derived PaCa 5061 pancreatic cancer cells were treated with gemcitabine (GEM; 0.5 µM) for 24 h or left untreated (control). (**A**,**C**,**E**) Relative KYNU mRNA expression was determined by quantitative real-time PCR (qRT-PCR) in Panc-1 (**A**), BxPC-3 (**C**), and PaCa 5061 (**E**) cells, respectively. Gemcitabine significantly increased KYNU mRNA expression in all three cell lines (Panc-1, *p* = 0.0028; BxPC-3, *p* = 0.0144; PaCa 5061, *p* = 0.0232; * *p* < 0.05; ** *p* < 0.01). (**B**,**D**,**F**) KYNU protein expression was assessed by Western blotting in the corresponding cell lines, with β-actin serving as a loading control. Gemcitabine treatment increased KYNU protein expression in all three cell lines, consistent with the transcriptional response.

In Panc-1 cells, gemcitabine exposure (0.5 µM) resulted in a pronounced induction of KYNU mRNA as determined by qRT-PCR (Figure 3A), reaching approximately an eight-fold increase relative to untreated controls. Cells remained morphologically intact during the treatment interval, indicating that KYNU upregulation occurred in viable, gemcitabine-exposed cultures. Consistent with transcript induction, immunoblotting demonstrated increased KYNU protein abundance following gemcitabine treatment (Figure 3B).

BxPC-3 cells and the primary PDAC line PaCa 5061 also showed increased KYNU transcript (Figure 3C,E) and protein levels upon gemcitabine treatment (Figure 3D,F), although the magnitude of induction was less pronounced than in Panc-1 cells. Collectively, these results are consistent with an association between gemcitabine-induced cellular stress and upregulation of KYNU expression in PDAC cells in vitro.

### 2.4. Interferon-γ and Hypoxia Induce KYNU Expression and Secretion in PDAC Cell Lines

To gain insight into upstream regulatory cues controlling KYNU expression, we next examined stress-related stimuli known to modulate enzymes of the tryptophan-kynurenine pathway. Interferon-γ (IFN-γ) is a well-established inducer of indoleamine-2,3-dioxygenase (IDO1) and tryptophan catabolism across multiple cellular contexts [30,31]. Moreover, IFN-γ-dependent regulation of KYNU activity has been reported in murine macrophages [32], but has not been systematically explored in PDAC models.

**Figure 4:**
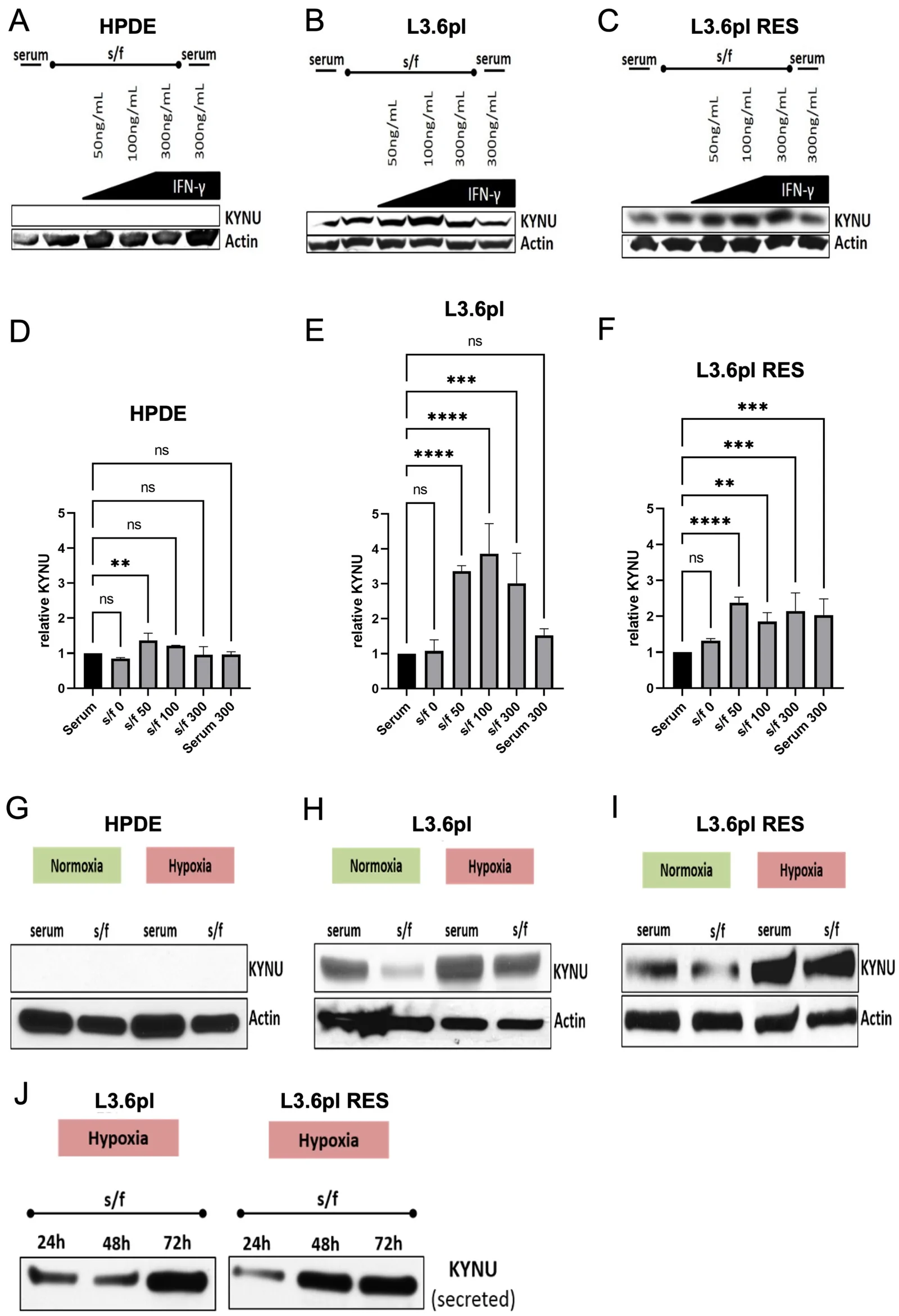
**IFN-γ and hypoxic stress induce KYNU expression and secretion in PDAC cells.** (**A**–**F**) HPDE (**A**,**D**), L3.6pl (**B**,**E**), and gemcitabine-resistant L3.6pl RES (**C**,**F**) cells were cultured under serum-free (s/f) conditions and treated with increasing concentrations of interferon-γ (IFN-γ; 50, 100, or 300 ng/mL), compared with serum conditions as the control. Intracellular KYNU protein levels were analyzed by Western blotting (**A**–**C**), with β-actin serving as a loading control, and KYNU mRNA expression was quantified by qRT-PCR (**D**–**F**). IFN-γ increased KYNU expression in L3.6pl and L3.6pl RES cells, whereas HPDE cells were unresponsive. (**G**–**I**) HPDE (**G**), L3.6pl (**H**), and L3.6pl RES (**I**) cells were cultured under serum-free conditions and exposed to normoxia or hypoxia (1% O_2_) for 24 h, compared with serum conditions as the control. Intracellular KYNU protein levels were assessed by Western blotting, with β-actin as a loading control. Hypoxia increased KYNU abundance, particularly in L3.6pl and L3.6pl RES cells. (**J**) L3.6pl and L3.6pl RES cells were cultured under serum-free conditions and exposed to hypoxia (1% O_2_) for 24, 48, or 72 h. Secreted KYNU in cell-free culture supernatants was analyzed by Western blotting and increased with prolonged hypoxic exposure. Data in (**D**–**F**) are presented as mean ± SD. Statistical significance is indicated as ns, not significant; **p < 0.01; *** *p* < 0.001; **** *p* < 0.0001.

HPDE, L3.6pl, and L3.6pl RES cells were cultured either with or without serum (s/f). For the latter, cells were serum-starved for 16 h, followed by IFN-γ stimulation for 24 h at increasing concentrations (50, 100, and 300 ng/mL). KYNU mRNA and protein levels were quantified by qRT-PCR and immunoblotting, respectively. In nonmalignant HPDE cells, IFN-γ did not induce KYNU expression above the low baseline at either the transcript or protein level (Figure 4A,D). In contrast, L3.6pl cells exhibited a reproducible increase in KYNU mRNA upon IFN-γ stimulation (approximately 3.5-fold; Figure 4E), with a corresponding increase in KYNU protein abundance (Figure 4B). Notably, serum-replete conditions attenuated IFN-γ responsiveness in L3.6pl cells, consistent with reduced cytokine-induced signaling in the presence of serum [33]. KYNU induction was even more pronounced in the chemoresistant L3.6pl RES line. Serum starvation alone increased KYNU mRNA levels in L3.6pl RES cells, and IFN-γ further augmented KYNU transcripts to approximately 5.5-fold over baseline (Figure 4F). Unlike parental L3.6pl cells, IFN-γ-mediated KYNU upregulation in L3.6pl RES cells remained robust under both serum-replete and serum-starved culture conditions. Western blot analyses closely mirrored the mRNA data, confirming IFN-γ-dependent induction of KYNU protein expression (Figure 4C). Collectively, these results indicate that KYNU is inducible by IFN-γ in PDAC cells, with enhanced responsiveness in the chemoresistant phenotype. The IFN-γ-dependent induction of KYNU protein expression in PDAC cell lines is accompanied by the dose-dependent KYNU secretion into the extracellular space (Supplementary Figure S2).

Beyond inflammatory signaling, we investigated whether hypoxia, an established feature of the hypovascularized PDAC microenvironment [6], modulates KYNU expression. Cells were cultured under hypoxic conditions (1% O_2_) for 24 h under serum-replete or serum-starved conditions. In HPDE cells, hypoxia did not induce detectable KYNU protein expression (Figure 4G). In contrast, both L3.6pl and L3.6pl RES cells showed increased intracellular KYNU protein levels under hypoxia (Figure 4H,I). Importantly, hypoxic culture also enhanced KYNU secretion, as analysis of cell-free supernatants demonstrated a time-dependent increase in secreted KYNU in PDAC cells during hypoxic incubation (24–72 h; Figure 4J).

Together, these findings identify IFN-γ and hypoxia as potent stress-associated stimuli that promote KYNU expression and secretion in PDAC cell models, supporting a link between microenvironmental stress adaptation and activation of KYNU in aggressive disease states.

### 2.5. High KYNU Expression Correlates with Poor Overall Survival in PDAC Patients

**Figure 5:**
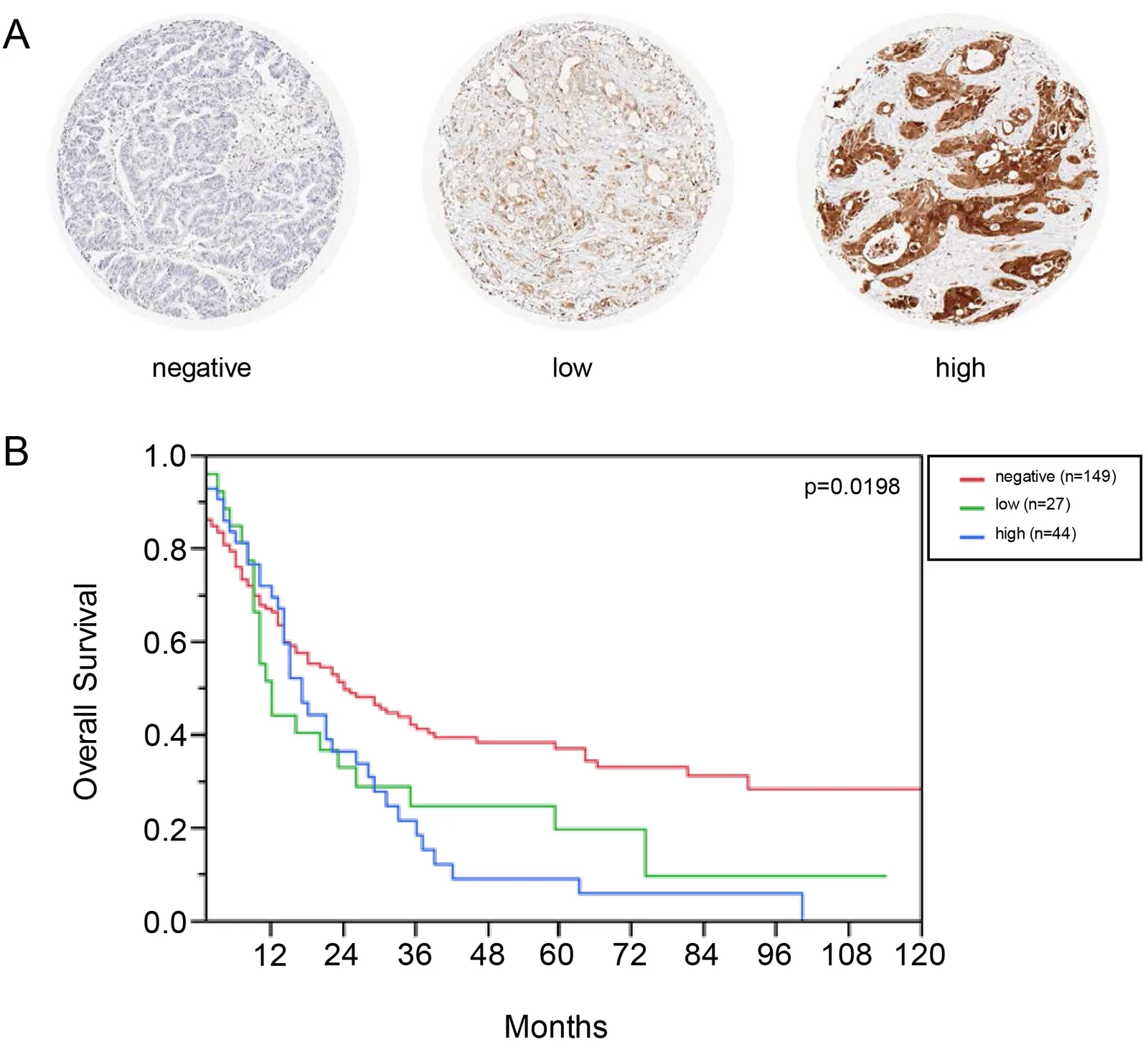
**Tissue microarray analysis of KYNU expression in PDAC patients.** A PDAC tissue microarray (*n* = 368) was analyzed by immunohistochemistry for KYNU protein expression. (**A**) Representative images illustrating cytoplasmic KYNU staining classified as negative, low, or high expression (original magnification, 100×). (**B**) Kaplan–Meier analysis of overall survival stratified by KYNU expression level. High KYNU expression was significantly associated with reduced overall survival in PDAC patients (*p* = 0.0198).

Among 368 analyzable pancreatic cancer specimens, KYNU expression was negative in 232 cases (63.0%), weak in 51 cases (13.9%), moderate in 50 cases (13.6%), and strong in 35 cases (9.5%). For subsequent analyses, tumors with moderate or strong KYNU staining were combined into a high-expression group, whereas tumors with weak staining were classified as low expression. Accordingly, KYNU expression was negative in 232 cases (63.0%), low in 51 cases (13.9%), and high in 85 cases (23.1%) (Supplementary Table S1). Representative immunohistochemical staining patterns are shown in Figure 5A. Associations between KYNU staining and clinicopathological tumor characteristics are summarized in Supplementary Table S1.

In survival analyses, high KYNU immunostaining was associated with significantly shorter overall survival in PDAC patients (*p* = 0.0494; Supplementary Figure S3). This association became more pronounced when tumors with high KYNU expression were compared with tumors showing low or negative KYNU expression, demonstrating significantly reduced overall survival among patients with high KYNU expression (*p* = 0.0198; Figure 5B).

In univariable Cox analysis, high KYNU expression was associated with poorer overall survival compared with negative expression (HR 1.63, 95% CI 1.11–2.40; *p* = 0.013; Supplementary Table S2). In the multivariable complete-case analysis, KYNU expression was not significantly associated with overall survival after adjustment for age, histologic grade, pT stage, and pN stage (overall *p* = 0.697; high vs. negative expression: adjusted HR 0.89, 95% CI 0.58–1.37; Supplementary Table S3).

### 2.6. Transcriptomic Profiling Confirms a Distinct Stress-Adapted Phenotype in Gemcitabine-Resistant L3.6pl RES Cells

For characterization of the molecular phenotype associated with acquired gemcitabine resistance, global transcriptomic profiling was performed in parental L3.6pl (WT) and gemcitabine-resistant L3.6pl RES cells using three biological replicates per condition. The complete gene-level raw read-count matrix for all WT and RES biological replicates is provided in Supplementary Table S4. Consistent with the KYNU-high phenotype, KYNU expression was higher in all three RES replicates than in the WT replicates (Supplementary Table S4).

**Figure 6:**
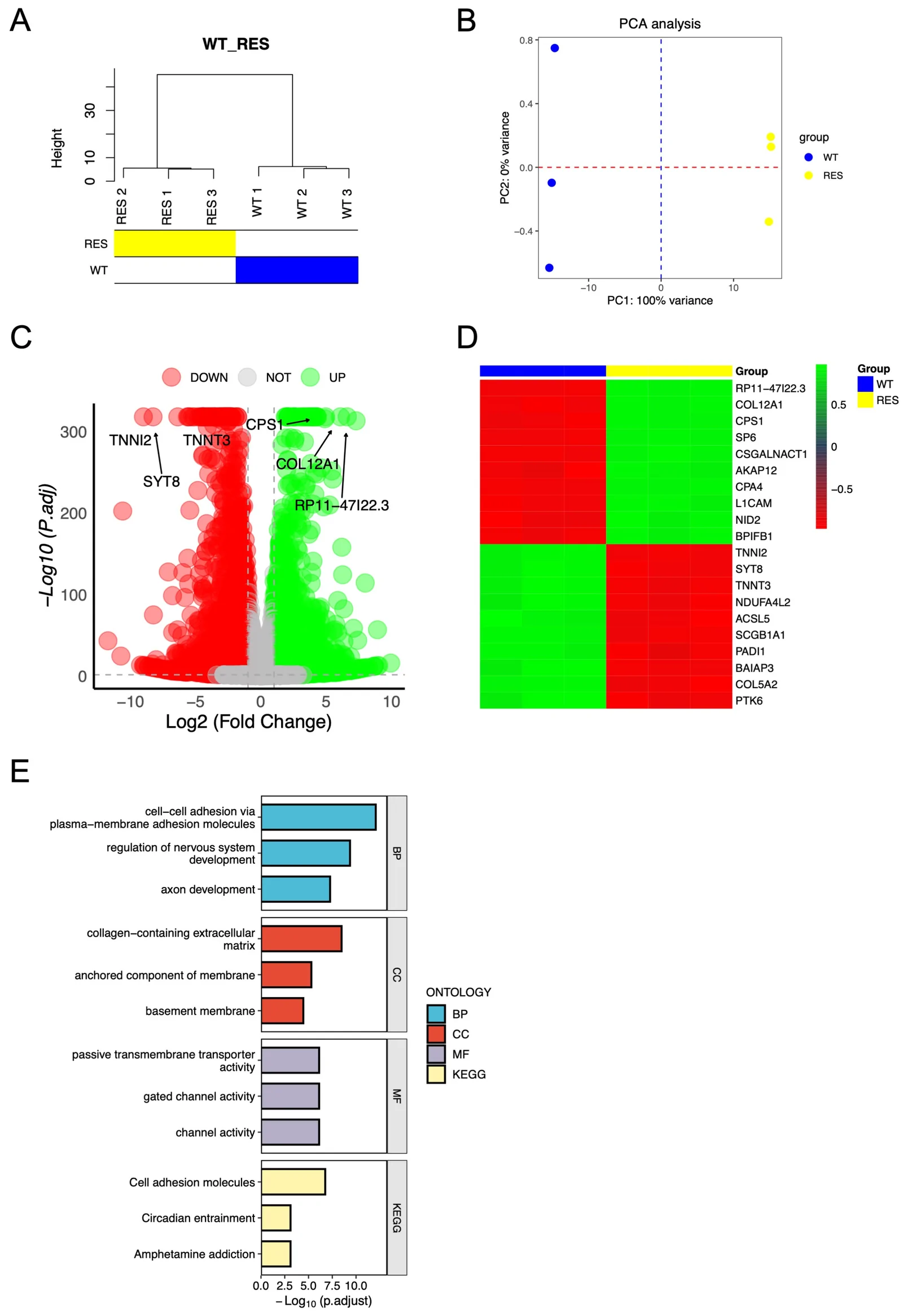
**Transcriptomic profiling reveals extensive molecular reprogramming in gemcitabine-resistant PDAC cells.** Global transcriptomic profiles of parental L3.6pl WT and gemcitabine-resistant L3.6pl RES cells were generated by RNA sequencing and compared using three biological replicates per condition, with WT cells serving as the reference. (**A**) Unsupervised hierarchical clustering separated WT from RES samples. (**B**) Principal component analysis demonstrated complete segregation of WT and RES cells. (**C**) Volcano plot showing differential gene expression in RES cells relative to WT cells. Genes with increased and decreased expression in RES cells are shown in green and red, respectively, whereas non-significantly altered genes are shown in gray. (**D**) Heatmap of selected differentially expressed genes showing consistent resistance-associated expression patterns across biological replicates. (**E**) Gene Ontology and KEGG pathway enrichment analysis of differentially expressed genes. The most significantly enriched terms within the Biological Process (BP), Cellular Component (CC), Molecular Function (MF), and KEGG categories are shown.

Hierarchical clustering and principal component analysis clearly separated WT from RES samples, confirming that L3.6pl RES cells represent a reproducible and transcriptionally distinct chemoresistant phenotype (Figure 6A,B).

Differential expression analysis revealed extensive transcriptional reprogramming in RES cells, with consistent expression patterns across biological replicates (Figure 6C,D). Functional enrichment analysis identified prominent alterations in cell adhesion, extracellular-matrix organization, and membrane-associated processes (Figure 6E). Resistance-associated genes were additionally enriched in response to xenobiotic stimulus (adjusted *p* = 0.0018), positive regulation of epithelial cell proliferation (adjusted *p* = 0.0024), response to interferon-γ (adjusted *p* = 0.0088), and response to hypoxia (adjusted *p* = 0.0134; Supplementary Table S5).

These findings are consistent with the stress-dependent KYNU induction observed in the preceding experiments. Collectively, the data indicate that acquired gemcitabine resistance is associated with coordinated remodeling of proliferation-, microenvironment-, and stress-response programs, providing a broader molecular context for the KYNU-high, treatment-adapted phenotype of L3.6pl RES cells.

### 2.7. Gene Set Enrichment Analysis Identifies Proliferative and Metabolic Adaptation Programs in Gemcitabine-Resistant L3.6pl RES Cells

**Figure 7:**
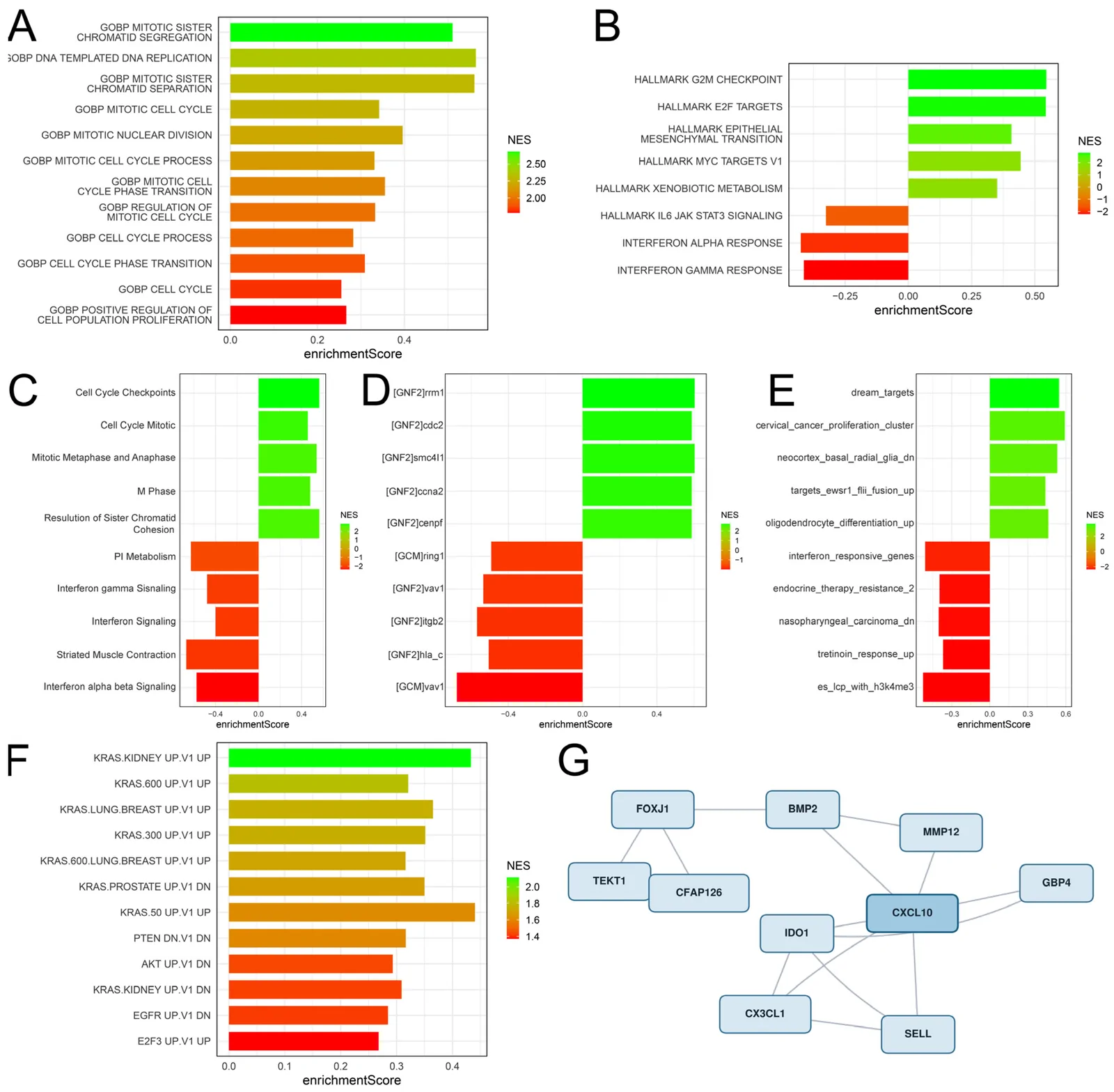
**Gene set enrichment analysis reveals proliferative, oncogenic, and immune-associated transcriptional programs in gemcitabine-resistant PDAC cells.** Pre-ranked gene set enrichment analysis (GSEA) was performed on genes ranked according to differential expression between gemcitabine-resistant L3.6pl RES and parental L3.6pl WT cells. Positive normalized enrichment scores (NESs) indicate enrichment in RES cells. (**A**) Gene Ontology Biological Process analysis highlighting enrichment of DNA replication, chromosome segregation, cell-cycle progression, and proliferation-associated pathways. (**B**) HALLMARK analysis identifying positive enrichment of G2/M checkpoint, E2F targets, MYC targets, epithelial–mesenchymal transition, and xenobiotic metabolism, alongside negative enrichment of interferon-α/γ response and IL6-JAK-STAT3 signaling. (**C**) Reactome pathway analysis showing enrichment of mitotic cell-cycle regulation and chromosome segregation, with negative enrichment of interferon signaling and metabolic pathways. (**D**) Enrichment of gene sets associated with cell-cycle regulators, including RRM1, CDC2 (CDK1), SMC4, CCNA2, and CENPF. (**E**) Chemical and genetic perturbation analysis highlighting DREAM complex targets and proliferation-associated signatures, alongside negatively enriched interferon-responsive gene sets. (**F**) Oncogenic signature analysis identifying enrichment of multiple KRAS-associated transcriptional signatures and differential enrichment of PTEN-, AKT-, EGFR-, and E2F3-related gene sets. (**G**) STRING-based protein–protein association network of differentially expressed genes, ranked by maximal clique centrality (MCC). CXCL10 emerged as the highest-ranked hub, with additional interconnected proteins including IDO1, CX3CL1, SELL, and GBP4, highlighting an immune-associated interaction network potentially relevant to the resistant phenotype.

To further characterize the transcriptional programs associated with acquired gemcitabine resistance, pre-ranked gene set enrichment analysis was performed using genes ordered according to their differential expression in L3.6pl RES cells relative to parental L3.6pl cells. Gene Ontology Biological Process analysis revealed enrichment of DNA replication, mitotic chromosome segregation, cell-cycle progression, and positive regulation of cell-population proliferation in RES cells (Figure 7A). Consistently, HALLMARK and Reactome analyses identified enrichment of G2/M checkpoint, E2F- and MYC-target, xenobiotic-metabolism, cell-cycle-checkpoint, and mitotic-progression programs (Figure 7B,C).

The proliferative phenotype was further supported by enrichment of co-expression signatures centered on ribonucleotide reductase catalytic subunit M1 (RRM1), cyclin-dependent kinase 1 (CDK1; CDC2), structural maintenance of chromosomes 4 (SMC4), cyclin A2 (CCNA2), and centromere protein F (CENPF), as well as DREAM-target and cancer-proliferation signatures (Figure 7D,E). Enrichment of the RRM1-associated signature is consistent with altered nucleotide metabolism and adaptation to gemcitabine. Together, these findings indicate that acquired gemcitabine resistance is associated with enhanced DNA-replication and mitotic programs. This proliferative transcriptional state is consistent with the reduced growth of L3.6pl RES cells following kynureninase knockdown.

Oncogenic gene-set analysis revealed broad transcriptional reprogramming of signatures derived from perturbations of KRAS proto-oncogene, GTPase (KRAS), phosphatase and tensin homolog (PTEN), AKT serine/threonine kinase signaling, epidermal growth factor receptor (EGFR), and E2F transcription factor 3 (E2F3) in RES cells (Figure 7F). Several independently defined KRAS-associated signatures were positively enriched, supporting remodeling of KRAS-associated transcriptional programs in the resistant cells.

To identify functionally connected components of the resistance-associated transcriptome, a protein–protein interaction network was generated from the differentially expressed genes and ranked using maximal clique centrality. C-X-C motif chemokine ligand 10 (CXCL10) was identified as the highest-ranked network node, while indoleamine 2,3-dioxygenase 1 (IDO1) formed an interaction module with CXCL10, C-X3-C motif chemokine ligand 1 (CX3CL1), selectin L (SELL), and guanylate-binding protein 4 (GBP4) (Figure 7G). The presence of IDO1, which catalyzes the initial rate-limiting step of the kynurenine pathway, together with interferon-responsive network components, links the chemoresistant transcriptional program to tryptophan metabolism and stress-associated signaling. Although KYNU was not among the highest-ranked network nodes, these findings provide additional evidence that the KYNU-high phenotype occurs within a broader kynurenine- and interferon-associated adaptation program.

## 3. Discussion

In this study, we demonstrate that KYNU is upregulated in a subset of PDAC and is associated with chemoresistance and poor patient outcomes. Interestingly, KYNU expression was inducible by various stress-associated stimuli, and functional analyses revealed that KYNU depletion impairs PDAC cell proliferation without significantly affecting migration.

Studies on KYNU expression in PDAC remain limited. However, our findings are consistent with the previous literature suggesting that KYNU expression in PDAC is associated with more aggressive tumor behavior and poor patient outcome [24]. León-Letelier et al. identified KYNU as a marker of tumor immunosuppression by demonstrating that Nrf2-induced KYNU overexpression in PDAC cells is associated with an immunosuppressive tumor phenotype and poor outcome, characterized by increased production of immunomodulatory metabolites, enhanced expression of immune checkpoint-related genes, and regulatory T-cell infiltration [24]. In addition, KYNU has been linked to chemoresistance and unfavorable tumor features in other tumor entities, including esophageal [34], lung [26], and breast cancer [35]. Mechanistically, increased KYNU activity may contribute to chemoresistance by promoting tumor stem cell properties [34,36] and generating immunomodulatory metabolites that favor an immunosuppressive tumor microenvironment [24,26]. For example, Lu et al. showed that KYNU is significantly upregulated in tumor cells and tissue of cisplatin-resistant esophageal cancer and that targeted downregulation of KYNU increases sensitivity to cisplatin, suppresses tumor stem cell-associated properties, and inhibits tumor growth [34]. Accordingly, upregulation of KYNU led to increased expression of stem cell markers such as SOX2, NANOG, and OCT4. In a bioinformatic multi-omics analysis of triple-negative breast cancer (TNBC), Lin et al. reported that kynurenine pathway genes, including KYNU, were upregulated and correlated with tumor progression and chemoresistance [35]. Immunofluorescence analyses further revealed nuclear accumulation of KYNU at sites of nuclear chromatin in TNBC cells [35], consistent with our data showing also a nuclear localization of KYNU in chemoresistant PDAC cells. The molecular role of nuclear KYNU needs further experimental validation in future studies. Nevertheless, to the best of our knowledge, the secretion of KYNU into the extracellular space was not observed in previous studies.

The gene-set enrichment analyses did not reveal an enrichment of any gene set annotated as tryptophan or kynurenine metabolism. Instead, DNA replication, cell cycle, and mitosis programs dominated in L3.6pl RES cells, including G2/M checkpoint, E2F, and MYC target signatures. The increased RRM1 expression within the enriched programs is particularly relevant, as elevated RRM1 expression has been described as a mechanism involved in acquired gemcitabine resistance in PDAC and other tumor models [37,38,39,40]. While E2F2 promotes gemcitabine resistance by upregulating RRM2, RRM1 is regulated as a target of E2F1 in resistant PDAC cells. Inhibition of the GSK3β-dependent Rb/E2F1 axis reduces RRM1 expression and restores sensitivity to gemcitabine [41,42]. Furthermore, MAPK-dependent regulation of RRM1/RRM2 expression has been described both through MEK inhibitor-induced degradation of RRM1 [43] and, in SMAD4-deficient PDAC cells, via an NFATc1/SMAD3/cJUN complex [44]. Our study identified a link to the kynurenine pathway in the network analysis, in which IDO1 formed an interferon-associated module together with CXCL10, CX3CL1, SELL and GBP4. Interferon- and cytokine-associated signaling networks have previously been linked to the activation of the IDO1/kynurenine axis in drug-resistant tumor cells. Campia et al. demonstrated that constitutive autocrine cytokine JAK/STAT signaling was associated with increased IDO1 expression and activity, as well as enhanced kynurenine production, in multidrug-resistant tumor cells [45]. Furthermore, downregulation of IDO1 in a tumor-cell-autonomous lung cancer model increased sensitivity to gemcitabine [46]. A further possible link arises from the enriched MYC-target signature, as MYC promoted tryptophan uptake and kynurenine formation in colon cancer cells [47]. Although there was therefore no pathway-wide upregulation of tryptophan metabolism and KYNU itself did not represent a central network node, the data suggest that the KYNU-high phenotype is embedded within a proliferative resistance program adapted to nucleotide metabolism, accompanied by interferon-IDO1-kynurenine signaling.

Considering our data, KYNU may be a potentially relevant therapeutic target, particularly with regard to the possible modulation of chemotherapy resistance in PDAC. However, pharmacological KYNU inhibitors have not yet been investigated in an oncological setting. The small-molecule inhibitors of KYNU available to date, including O-methoxybenzoylalanine (a kynurenine analogue) [48] and benserazide hydrochloride [49], have been studied primarily in non-oncological disease models or in the context of kynurenine metabolism outside the field of tumor biology. In a study by Yang et al., the overexpression of KYNU in CAR-T cells led to increased kynurenine degradation in the tumor microenvironment. This attenuated kynurenine-mediated impairments in T-cell function and enhanced the antitumor activity of CAR-T cells in preclinical models [50]. These results underscore the importance of kynurenine metabolism in tumor-associated immunosuppression, but also demonstrate that the therapeutic role of KYNU is likely to be context-dependent. It therefore remains to be seen whether direct pharmacological inhibition of KYNU can actually mitigate KYNU-associated tumor progression or chemotherapy resistance in PDAC.

In summary, KYNU is upregulated in a subset of PDAC and is associated with a therapy-adapted, aggressive phenotype. Its association with poor survival and preferential secretion by chemoresistant cells supports KYNU as a potential prognostic and treatment-response biomarker in PDAC.

## 4. Materials and Methods

### 4.1. Cell Lines and Cell Culture

The human pancreatic ductal adenocarcinoma cell lines L3.6pl (RRID: CVCL_0384), PANC-1 (RRID: CVCL_0480), BxPC-3 (RRID: CVCL_0186), MIA PaCa-2 (RRID: CVCL_0428), PaCa 5061 (RRID: CVCL_C886), PaCa 5072 (RRID: CVCL_C887), and PaCa 5156 (RRID: CVCL_LM69), as well as the human pancreatic ductal epithelial cell line HPDE (RRID: CVCL_4376), were used in this study. L3.6pl and HPDE cells were kindly provided by Prof. Christiane Bruhns. PANC-1 (CRL-1469), BxPC-3 (CRL-1687), and MIA PaCa-2 (CRL-1420) cells were obtained from the American Type Culture Collection (ATCC). The patient-derived PDAC cell lines PaCa 5061, PaCa 5072, and PaCa 5156 were established at the University Medical Center Hamburg–Eppendorf (UKE), Hamburg, Germany.

Human pancreatic ductal epithelial (HPDE) cells were cultured in keratinocyte serum-free medium (K-SFM; Invitrogen, Carlsbad, CA, USA)). The establishment and characterization of HPDE cells have been described previously [51]. The pancreatic ductal adenocarcinoma (PDAC) cell line L3.6pl was maintained in RPMI 1640 (Invitrogen) as reported [52]. A gemcitabine-resistant derivative (L3.6pl RES) was generated from parental L3.6pl cells by continuous exposure to increasing concentrations of gemcitabine for 6 months, up to a final concentration of 2 µmol/L for 30 days, as described previously [53]. PANC-1 and BxPC-3 cells were cultured in Dulbecco’s modified Eagle medium (DMEM) [54,55].

Primary PDAC cell lines were established from PDAC patient specimens obtained at the Department of General, Visceral, Transplant and Thoracic Surgery, University Medical Center Hamburg–Eppendorf, and propagated in tumor medium (TUM) as previously described [56].

Unless stated otherwise, all culture media were supplemented with 10% fetal calf serum (FCS; Lonza, Basel, Switzerland) and penicillin/streptomycin (200 IU/mL; Sigma-Aldrich, St. Louis, MO, USA). Cells were maintained at 37 °C in a humidified incubator with 5% CO_2_.

For serum-free conditions, cells were grown to 70–80% confluence, washed as appropriate, and the serum-containing medium was replaced by serum-free medium 16 h prior to the start of the experiment. Hypoxic culture conditions were performed at 1% O_2_. For cytokine stimulation experiments, interferon-γ (IFN-γ; Invitrogen) was added to the culture medium at final concentrations of 50, 100, or 300 ng/mL.

### 4.2. γ-Retrovirus-Mediated shRNA Knockdown of KYNU in L3.6pl RES Cells

γ-Retroviral particles encoding two independent KYNU-targeting shRNAs (constructs KD-1 and KD-2) to avoid off-target effects, or a non-targeting control shRNA (OriGene, Rockville, MD, USA) were produced in HEK293T cells. For virus production, HEK293T cells were co-transfected with plasmids encoding Gag/Pol, GALV/Env, and the respective shRNA vector using a calcium phosphate precipitation method. Briefly, plasmid DNA was mixed with 2.5 M CaCl_2_ and added to 2× HEPES-buffered saline (HBS) to form DNA-calcium phosphate precipitates. The transfection mixture was added dropwise to HEK293T cultures maintained in chloroquine-containing medium while gently mixing to ensure even distribution. After 6 h, the medium was replaced with fresh DMEM.

Virus-containing supernatants were collected at 12 h and 24 h after the medium change and stored at −80 °C until use. For transduction, 1 mL of viral supernatant was added to 70–80% confluent L3.6pl RES cells in six-well plates in the presence of polybrene. Plates were centrifuged for 1 h at 2200 rpm using a swing-out rotor (spinoculation). The following day, the medium was replaced with RPMI 1640. Stable knockdown populations/clones were selected by adding puromycin (1 µg/mL) to the culture medium.

### 4.3. Cell Proliferation Assay

Cell proliferation/viability was assessed using an MTT-based colorimetric assay according to the manufacturer’s instructions (Promega, Madison, WI, USA) in 96-well plates. Briefly, cells were seeded at defined densities, and MTT reagent was added at 24-h intervals. After incubation to allow metabolic conversion of the substrate, absorbance was measured at 490 nm using a FLUOstar Omega microplate reader (BMG Labtech, Ortenberg, Germany). Measurements were performed in technical quadruplicates. For data normalization, absorbance values were normalized to the baseline (time 0) absorbance determined at the start of each experiment. Each experiment was repeated at least three times independently.

### 4.4. Colony Formation Assay

Cells were seeded into 6-well plates at a density of 2 × 10^3^ cells per well in triplicate for each of the three groups. Every 48 h, the media were replaced with fresh medium containing gemcitabine at a final concentration of 2 μM, and cells were cultured for an additional 14 days. Subsequently, cells were fixed with PFA (4%) for 15 min at RT, washed 3× with PBS, stained with crystal violet (Sigma-Aldrich) for 15 min, and washed again 3× with PBS. Images were captured and processed using Fiji (ImageJ version 2.16.0/1.54p). The colony-covered area was quantified as a percentage of the total 6-well area. Data are presented as mean ± SD from three replicate wells per group. Ordinary one-way ANOVA was used for comparisons among groups. * *p* < 0.05 and *** *p* < 0.001 were considered statistically significant.

### 4.5. Gemcitabine Dose–Response and WST-1 Assays

Gemcitabine sensitivity was assessed in L3.6pl WT, gemcitabine-resistant sh-Control, and two KYNU-knockdown populations KD-1 and KD-2. Cells were seeded in 96-well plates at 5 × 10^3^ cells/well in 90 µL complete medium and treated with gemcitabine at final concentrations of 0, 0.003, 0.01, 0.03, 0.1, 0.3, 1, 3, 10, and 30 µM for 48 h. Cell viability/metabolic activity was determined using the WST-1 assay after 3 h of incubation with WST-1 reagent. Absorbance was measured at 440 nm with a 600-nm reference measurement, and corrected values were calculated by subtracting the 600-nm signal from the corresponding 440-nm absorbance. Three independent experiments were performed, each with technical duplicates. Technical replicates were averaged before analysis, and values were normalized to the corresponding untreated control, defined as 100%. Dose–response curves were generated from normalized viability values. Because the resistant control and KYNU-knockdown populations did not consistently reach 50% viability within the tested concentration range, drug sensitivity was quantified primarily by area under the dose–response curve (AUC), calculated across 0.003–30 µM gemcitabine using log10-transformed concentrations and the trapezoidal method.

### 4.6. Cell Migration Assay

Cell migration was assessed using a Transwell assay. Briefly, cells were resuspended at 5 × 10^5^ cells/mL in DMEM containing 0.1% fetal bovine serum (FBS) and seeded into the upper chamber of Transwell inserts (8-µm pore size; Costar Transwell Permeable Supports, Corning Incorporated, Corning, NY, USA). The lower chambers were filled with DMEM supplemented with 10% FBS to serve as a chemoattractant. After 24 h of incubation, cells that had migrated to the underside of the membrane were processed for quantification either by fixation and staining followed by counting in four randomly selected microscopic fields per insert, or trypsinization and subsequent cell counting.

### 4.7. RNA Isolation and Quantitative Real-Time Reverse-Transcription PCR (qRT-PCR)

Total RNA was isolated using the RNeasy Mini Kit (Qiagen, Hilden, Germany) according to the manufacturer’s protocol. RNA was reverse-transcribed into cDNA via Transcriptor Reverse Transcriptase (Roche, Mannheim, Germany) and oligo (dT) primers following the supplier’s instructions. Quantitative real-time PCR (qRT-PCR) was performed using Maxima SYBR Green/ROX qPCR Master Mix (Thermo Fisher Scientific, Waltham, MA, USA), 0.3 μM each forward and reverse primer, and 750 ng of cDNA per reaction on a LightCycler 480 system (Roche). Cycling conditions were 95 °C for 10 min, followed by 45 cycles of 95 °C for 15 s and 60 °C for 1 min. CT values were determined via LightCycler 480 software version 1.5 (Roche). Each sample was analyzed in technical duplicate, and quantitative values were normalized to GAPDH expression via the 2^−ΔΔCT^ method. All experiments were repeated at least twice for consistency.

### 4.8. Western Blot

Viable cells were lysed in RIPA buffer (Sigma-Aldrich) supplemented with 1% protease inhibitor. For analysis of secreted proteins, cell culture supernatants were collected, cleared by filtration (0.22 µm), and concentrated by centrifugation using ultrafiltration units (molecular weight cutoff ≥3000 Da) at 5000× *g*.

Total protein concentrations were determined using the BCA protein assay (Thermo Fisher Scientific). Equal amounts of protein (30 µg per lane) were mixed with Laemmli sample buffer, boiled for 5 min, and separated by SDS-PAGE under reducing conditions using 4–15% Mini-PROTEAN TGX gels (Bio-Rad, Hercules, CA, USA). Proteins were transferred to nitrocellulose membranes (Thermo Fisher Scientific). Membranes were blocked in 1× Carbo-Free Blocking Solution (Vector Laboratories, Newark, CA, USA; supplied by Biozol, Hamburg, Germany) prepared in TBS-T and incubated overnight at 4 °C with primary antibodies against kynureninase (KYNU) (clone 589731, R&D Systems, Minneapolis, MN, USA; 1:500, or ab96365, Abcam, Cambridge, UK; 1:1000). α-Tubulin and/or β-actin were used as loading controls.

After washing five times with TBS-T, membranes were incubated with appropriate HRP-conjugated secondary antibodies, and protein bands were detected by enhanced chemiluminescence (ECL; GE Healthcare, Little Chalfont, UK).

### 4.9. Immunofluorescence

Cells were cultured on coverslips, fixed with 4% formaldehyde for 10 min, washed with PBS, and afterwards blocked for 1 h in 1% BSA/PBS-T at RT. The cells were then incubated overnight at 4 °C with an anti-kynureninase (KYNU) primary antibody (clone 589731; R&D Systems). After washing with PBS, cells were incubated for 1 h at room temperature with a fluorescein-conjugated secondary antibody (Invitrogen). Following additional PBS washes, fluorescence images were acquired using a Leica confocal microscope (Leica Microsystems, Wetzlar, Germany).

Protein bands were quantified using the imaging system. Only non-saturated images within the linear range were used for quantification. Band intensities were measured using Fiji (ImageJ version 2.16.0/1.54p). Images were converted to 8-bit grayscale, background was subtracted using the rolling-ball method (radius = 50 pixels), and the images were inverted. A fixed-size rectangular region of interest (ROI) was used to select each band, and the integrated density (IntDen) was measured. When necessary, background signal was measured from a blank area of the same size and subtracted from the band IntDen. The net intensity of the target protein was normalized to that of the loading control. The control or RES group was set as 100%, and data are presented as relative band intensity (% of control/RES). All bands were measured using identical parameters.

### 4.10. Tissue Microarray (TMA)

A pancreatic cancer tissue microarray (TMA) comprising 368 formalin-fixed, paraffin-embedded (FFPE) PDAC specimens from patients who underwent surgical resection at the University Medical Center Hamburg–Eppendorf was analyzed. The study was approved by the local ethics committee (WF-049/09, PV3652) and conducted in accordance with the Declaration of Helsinki. TMA construction was performed as described previously [57]. Tumors were graded and staged according to WHO and UICC criteria, respectively. Fresh TMA sections were processed in a single staining run to minimize batch effects. Antigen retrieval was performed by autoclaving slides at 121 °C for 5 min in retrieval buffer (pH 2.0; BioGenex, Fremont, CA, USA). Sections were incubated for 60 min at 37 °C with an anti-kynureninase (KYNU) primary antibody (clone 589731; R&D Systems) at a 1:100 dilution, followed by signal detection using the EnVision system (Dako, Glostrup, Denmark). For tumor tissues, the staining intensity of tumor cells was semi-quantitatively recorded (0 = negative, 1+ = weak, 2+ = moderate, or 3+ = strong). The presence of unequivocal KYNU positivity in stromal cells was particularly required to classify a tumor as negative.

### 4.11. SILAC (Stable Isotope Labeling by Amino Acids in Cell Culture)

L3.6pl RES and parental L3.6pl cells were maintained in SILAC RPMI 1640 medium (Thermo Fisher Scientific) supplemented with 10% dialyzed FCS (Thermo Fisher Scientific) and penicillin/streptomycin (200 IU/mL; Invitrogen) at 37 °C in a humidified atmosphere with 5% CO2. For metabolic labeling, gemcitabine-resistant L3.6pl RES cells (continuously cultured in the presence of 2 µM gemcitabine) were grown in “heavy” SILAC medium containing 13C6-Arginine and 13C6-Lysine, whereas chemosensitive parental L3.6pl cells were cultured in the corresponding “light” (unlabeled) medium, according to the manufacturer’s instructions. Cells were propagated under labeling conditions for a minimum of eight passages/split cycles to ensure efficient incorporation of labeled amino acids prior to secretome collection and downstream proteomic analyses.

### 4.12. Mass Spectrometry (MS)

Following tryptic digestion, peptides derived from “light” and “heavy” labeled samples were mixed as indicated and analyzed by liquid chromatography-tandem mass spectrometry (LC-MS/MS) to verify efficient isotope incorporation and to quantify relative protein abundance between conditions. Raw MS data were processed using MaxQuant software (version 2.0.0, Max Planck Institute of Biochemistry, Martinsried, Germany) [58]. Protein identification was performed by database searching of MS/MS spectra, and relative quantification was calculated from the intensity ratios of corresponding SILAC peptide pairs.

For downstream candidate selection, proteins were considered differentially abundant if they met a minimum fold-change threshold of ≥5 and were statistically significant (*p* ≤ 0.05).

### 4.13. Transcriptomic Analysis and Pathway Enrichment

RNA sequencing was performed at the Leibniz Institute of Virology (LIV, Hamburg, Germany). Differential gene expression analysis comparing L3.6pl cells with L3.6pl RES cells was performed using the R package DESeq2 [59]. Each cell line was analyzed in biological triplicates. Genes with an absolute log2 fold change > 1 and an adjusted *p*-value < 0.05 were considered significantly differentially expressed. The contrast was defined as L3.6pl RES versus L3.6pl, such that positive log2 fold-change values indicated higher expression in L3.6pl RES cells relative to L3.6pl cells. Gene Ontology (GO) and Kyoto Encyclopedia of Genes and Genomes (KEGG) pathway enrichment analyses were performed using the R package clusterProfiler [60,61,62], with an adjusted *p*-value < 0.05 considered significant. Gene set enrichment analysis (GSEA) was performed using the Molecular Signatures Database (MSigDB) Hallmark (H) and curated canonical pathway (C2) gene sets [63,64]. Genes were ranked according to differential expression between L3.6pl RES and L3.6pl cells, and normalized enrichment scores (NESs) were calculated for each gene set. Protein–protein interaction (PPI) networks were generated from the top 150 differentially expressed genes using the STRING database with a high-confidence interaction score cutoff of ≥0.700 [65]. The resulting networks were visualized using Cytoscape v3.10.5 [66].

### 4.14. Statistical Analysis

All experiments were performed at least twice independently. Unless stated otherwise, data are presented as mean ± SEM. For comparisons between two independent groups, statistical significance was assessed using a two-tailed, unpaired Student’s *t*-test. A *p*-value < 0.05 was considered statistically significant. Overall survival was analyzed using the Kaplan–Meier method, and differences between survival curves were evaluated by the log-rank (Mantel–Cox) test. For survival analyses, *p* < 0.05 was considered significant. Associations with overall survival were evaluated using univariable and multivariable Cox proportional hazards regression. Univariable analyses used all available cases for each variable (KYNU expression, histologic grade, pathologic T stage, pathologic N stage, and age). The multivariable analysis included patients with complete data (*n* = 155), with all five variables entered simultaneously. Hazard ratios and corresponding 95% confidence intervals were reported. AUC values from the three independent experiments were compared between resistant sh-Control and KYNU-knockdown cells using paired two-tailed *t*-tests. Data are presented as mean ± SD, and *p* < 0.05 was considered statistically significant. Statistical analyses were performed using GraphPad Prism version 9.5.1 (GraphPad Software, La Jolla, CA, USA), JMP version 18 (SAS Institute, Cary, NC, USA), and R version 4.3.0 (R Core Team, 2023).

## Data Availability

The original contributions presented in this study are included in the article and the Supplementary Materials. Further inquiries can be directed to the corresponding authors.

## Supplementary Materials

The following supporting information can be downloaded at: https://www.mdpi.com/article/10.3390/ijms27219820/s1.

## References

[1] Stoop T.F., Javed A.A., Oba A., Koerkamp B.G., Seufferlein T., Wilmink J.W., Besselink M.G. (2025). Pancreatic cancer. Lancet.

[2] Kolbeinsson H.M., Chandana S., Wright G.P., Chung M. (2023). Pancreatic Cancer: A Review of Current Treatment and Novel Therapies. J. Investig. Surg..

[3] Ponz de Leon Pisani R., Archibugi L., Lazzano P., Bina N., Vanella G., Lauri G., Tacelli M., Apadula L., Tamburrino D., Aleotti F. (2025). Diagnostic delay at diagnosis and time-to-treatment influence overall survival of pancreatic cancer patients. Dig. Liver Dis..

[4] Mizrahi J.D., Surana R., Valle J.W., Shroff R.T. (2020). Pancreatic cancer. Lancet.

[5] Thakur R., Mullen N.J., Mehla K., Singh P.K. (2025). Tumor-stromal metabolic crosstalk in pancreatic cancer. Trends Cell Biol..

[6] Ryan D.P., Hong T.S., Bardeesy N. (2014). Pancreatic adenocarcinoma. N. Engl. J. Med..

[7] Hilmi M., Delaye M., Muzzolini M., Nicolle R., Cros J., Hammel P., Cardot-Ruffino V., Neuzillet C. (2023). The immunological landscape in pancreatic ductal adenocarcinoma and overcoming resistance to immunotherapy. Lancet Gastroenterol. Hepatol..

[8] Zeng S., Pottler M., Lan B., Grutzmann R., Pilarsky C., Yang H. (2019). Chemoresistance in Pancreatic Cancer. Int. J. Mol. Sci..

[9] Burris H.A., Moore M.J., Andersen J., Green M.R., Rothenberg M.L., Modiano M.R., Cripps M.C., Portenoy R.K., Storniolo A.M., Tarassoff P. (1997). Improvements in survival and clinical benefit with gemcitabine as first-line therapy for patients with advanced pancreas cancer: A randomized trial. J. Clin. Oncol..

[10] Von Hoff D.D., Ervin T., Arena F.P., Chiorean E.G., Infante J., Moore M., Seay T., Tjulandin S.A., Ma W.W., Saleh M.N. (2013). Increased survival in pancreatic cancer with nab-paclitaxel plus gemcitabine. N. Engl. J. Med..

[11] Conroy T., Desseigne F., Ychou M., Bouche O., Guimbaud R., Becouarn Y., Adenis A., Raoul J.L., Gourgou-Bourgade S., de la Fouchardiere C. (2011). FOLFIRINOX versus gemcitabine for metastatic pancreatic cancer. N. Engl. J. Med..

[12] Conroy T., Pfeiffer P., Vilgrain V., Lamarca A., Seufferlein T., O’Reilly E.M., Hackert T., Golan T., Prager G., Haustermans K. (2023). Pancreatic cancer: ESMO Clinical Practice Guideline for diagnosis, treatment and follow-up. Ann. Oncol..

[13] Klein-Brill A., Amar-Farkash S., Lawrence G., Collisson E.A., Aran D. (2022). Comparison of FOLFIRINOX vs Gemcitabine Plus Nab-Paclitaxel as First-Line Chemotherapy for Metastatic Pancreatic Ductal Adenocarcinoma. JAMA Netw. Open.

[14] Mastrantoni L., Chiaravalli M., Spring A., Beccia V., Di Bello A., Bagala C., Bensi M., Barone D., Trovato G., Caira G. (2024). Comparison of first-line chemotherapy regimens in unresectable locally advanced or metastatic pancreatic cancer: A systematic review and Bayesian network meta-analysis. Lancet Oncol..

[15] Sarvepalli D., Rashid M.U., Rahman A.U., Ullah W., Hussain I., Hasan B., Jehanzeb S., Khan A.K., Jain A.G., Khetpal N. (2019). Gemcitabine: A Review of Chemoresistance in Pancreatic Cancer. Crit. Rev. Oncog..

[16] Li Z., Zhang H. (2016). Reprogramming of glucose, fatty acid and amino acid metabolism for cancer progression. Cell. Mol. Life Sci..

[17] Lieu E.L., Nguyen T., Rhyne S., Kim J. (2020). Amino acids in cancer. Exp. Mol. Med..

[18] Shuvalov O., Daks A., Fedorova O., Petukhov A., Barlev N. (2021). Linking Metabolic Reprogramming, Plasticity and Tumor Progression. Cancers.

[19] Honigova K., Navratil J., Peltanova B., Polanska H.H., Raudenska M., Masarik M. (2022). Metabolic tricks of cancer cells. Biochim. Biophys. Acta Rev. Cancer.

[20] Sniegowski T., Korac K., Bhutia Y.D., Ganapathy V. (2021). SLC6A14 and SLC38A5 Drive the Glutaminolysis and Serine-Glycine-One-Carbon Pathways in Cancer. Pharmaceuticals.

[21] Endicott M., Jones M., Hull J. (2021). Amino acid metabolism as a therapeutic target in cancer: A review. Amino Acids.

[22] Phillips R.S. (2014). Structure and mechanism of kynureninase. Arch. Biochem. Biophys..

[23] Xue C., Li G., Zheng Q., Gu X., Shi Q., Su Y., Chu Q., Yuan X., Bao Z., Lu J. (2023). Tryptophan metabolism in health and disease. Cell Metab..

[24] Leon-Letelier R.A., Abdel Sater A.H., Chen Y., Park S., Wu R., Irajizad E., Dennison J.B., Katayama H., Vykoukal J.V., Hanash S. (2023). Kynureninase Upregulation Is a Prominent Feature of NFR2-Activated Cancers and Is Associated with Tumor Immunosuppression and Poor Prognosis. Cancers.

[25] Fahrmann J.F., Tanaka I., Irajizad E., Mao X., Dennison J.B., Murage E., Casabar J., Mayo J., Peng Q., Celiktas M. (2022). Mutational Activation of the NRF2 Pathway Upregulates Kynureninase Resulting in Tumor Immunosuppression and Poor Outcome in Lung Adenocarcinoma. Cancers.

[26] Cai L., Rogers T.J., Mousavi Jafarabad R., Vu H., Yang C., Novaresi N., Galan-Cobo A., Girard L., Ostrin E.J., Fahrmann J.F. (2025). High KYNU Expression Is Associated with Poor Prognosis, KEAP1/STK11 Mutations, and Immunosuppressive Metabolism in Patient-Derived but Not Murine Lung Adenocarcinomas. Cancers.

[27] Li Y., Wang M., Zhao L., Liang C., Li W. (2023). KYNU-related transcriptome profile and clinical outcome from 2994 breast tumors. Heliyon.

[28] Inada J., Okuno E., Kimura M., Kido R. (1984). Intracellular localization and characterization of 3-hydroxykynureninase in human liver. Int. J. Biochem..

[29] Walsh H.A., Botting N.P. (2002). Purification and biochemical characterization of some of the properties of recombinant human kynureninase. Eur. J. Biochem..

[30] Taylor M.W., Feng G.S. (1991). Relationship between interferon-gamma, indoleamine 2,3-dioxygenase, and tryptophan catabolism. FASEB J..

[31] Yoshida R., Imanishi J., Oku T., Kishida T., Hayaishi O. (1981). Induction of pulmonary indoleamine 2,3-dioxygenase by interferon. Proc. Natl. Acad. Sci. USA.

[32] Alberati-Giani D., Ricciardi-Castagnoli P., Kohler C., Cesura A.M. (1996). Regulation of the kynurenine metabolic pathway by interferon-gamma in murine cloned macrophages and microglial cells. J. Neurochem..

[33] Shi Y., Wang R., Sharma A., Gao C., Collins M., Penn L., Mills G.B. (1997). Dissociation of cytokine signals for proliferation and apoptosis. J. Immunol..

[34] Lu Y., Zhao X., Yuan M., Zhao M., Liu K., Zhang M., Qiu X., Yu X., Liu X., Wei D. (2024). KYNU Expression Promotes Cisplatin Resistance in Esophageal Cancer. J. Cancer.

[35] Lin M., Zhou J., Xiao J., Li C., Mo Y., Liu Y., Xiao Y., Huang J., Feng X. (2025). Integrating multi-omics data of Triple-Negative Breast Cancer to explore the role of Kynurenine pathway and KYNU as a therapeutic target. Biochem. Biophys. Res. Commun..

[36] Pan X., Wang W., Wang Y., Gu J., Ma X. (2025). M2 macrophage-secreted KYNU promotes stemness remodeling and malignant behavior in endometrial cancer via the SOD2-mtROS-ERO1alpha-UPR(ER) axis. J. Exp. Clin. Cancer Res..

[37] Nakahira S., Nakamori S., Tsujie M., Takahashi Y., Okami J., Yoshioka S., Yamasaki M., Marubashi S., Takemasa I., Miyamoto A. (2007). Involvement of ribonucleotide reductase M1 subunit overexpression in gemcitabine resistance of human pancreatic cancer. Int. J. Cancer.

[38] Bergman A.M., Eijk P.P., Ruiz van Haperen V.W., Smid K., Veerman G., Hubeek I., van den Ijssel P., Ylstra B., Peters G.J. (2005). In vivo induction of resistance to gemcitabine results in increased expression of ribonucleotide reductase subunit M1 as the major determinant. Cancer Res..

[39] Jordheim L.P., Seve P., Tredan O., Dumontet C. (2011). The ribonucleotide reductase large subunit (RRM1) as a predictive factor in patients with cancer. Lancet Oncol..

[40] Zhou J., Zhang L., Zheng H., Ge W., Huang Y., Yan Y., Zhou X., Zhu W., Kong Y., Ding Y. (2020). Identification of chemoresistance-related mRNAs based on gemcitabine-resistant pancreatic cancer cell lines. Cancer Med..

[41] Liu Q., Song C., Li J., Liu M., Fu L., Jiang J., Zeng Z., Zhu H. (2022). E2F2 enhances the chemoresistance of pancreatic cancer to gemcitabine by regulating the cell cycle and upregulating the expression of RRM2. Med. Oncol..

[42] Uehara M., Domoto T., Takenaka S., Bolidong D., Takeuchi O., Miyashita T., Minamoto T. (2020). Glycogen synthase kinase-3beta participates in acquired resistance to gemcitabine in pancreatic cancer. Cancer Sci..

[43] Vena F., Li Causi E., Rodriguez-Justo M., Goodstal S., Hagemann T., Hartley J.A., Hochhauser D. (2015). The MEK1/2 Inhibitor Pimasertib Enhances Gemcitabine Efficacy in Pancreatic Cancer Models by Altering Ribonucleotide Reductase Subunit-1 (RRM1). Clin. Cancer Res..

[44] Hasselluhn M.C., Schlosser D., Versemann L., Schmidt G.E., Ulisse M., Oschwald J., Zhang Z., Hamdan F., Xiao H., Kopp W. (2024). An NFATc1/SMAD3/cJUN Complex Restricted to SMAD4-Deficient Pancreatic Cancer Guides Rational Therapies. Gastroenterology.

[45] Campia I., Buondonno I., Castella B., Rolando B., Kopecka J., Gazzano E., Ghigo D., Riganti C. (2015). An Autocrine Cytokine/JAK/STAT-Signaling Induces Kynurenine Synthesis in Multidrug Resistant Human Cancer Cells. PLoS ONE.

[46] Maleki Vareki S., Chen D., Di Cresce C., Ferguson P.J., Figueredo R., Pampillo M., Rytelewski M., Vincent M., Min W., Zheng X. (2015). IDO Downregulation Induces Sensitivity to Pemetrexed, Gemcitabine, FK866, and Methoxyamine in Human Cancer Cells. PLoS ONE.

[47] Venkateswaran N., Lafita-Navarro M.C., Hao Y.H., Kilgore J.A., Perez-Castro L., Braverman J., Borenstein-Auerbach N., Kim M., Lesner N.P., Mishra P. (2019). MYC promotes tryptophan uptake and metabolism by the kynurenine pathway in colon cancer. Genes Dev..

[48] Chiarugi A., Carpenedo R., Molina M.T., Mattoli L., Pellicciari R., Moroni F. (1995). Comparison of the neurochemical and behavioral effects resulting from the inhibition of kynurenine hydroxylase and/or kynureninase. J. Neurochem..

[49] Zhang Y., Wang L., Ren W. (2022). Blast-related traumatic brain injury is mediated by the kynurenine pathway. Neuroreport.

[50] Yang Q., Hao J., Chi M., Wang Y., Xin B., Huang J., Lu J., Li J., Sun X., Li C. (2022). Superior antitumor immunotherapy efficacy of kynureninase modified CAR-T cells through targeting kynurenine metabolism. Oncoimmunology.

[51] Ouyang H., Mou L., Luk C., Liu N., Karaskova J., Squire J., Tsao M.S. (2000). Immortal human pancreatic duct epithelial cell lines with near normal genotype and phenotype. Am. J. Pathol..

[52] Bruns C.J., Harbison M.T., Kuniyasu H., Eue I., Fidler I.J. (1999). In vivo selection and characterization of metastatic variants from human pancreatic adenocarcinoma by using orthotopic implantation in nude mice. Neoplasia.

[53] Güngör C., Zander H., Effenberger K.E., Vashist Y.K., Kalinina T., Izbicki J.R., Yekebas E., Bockhorn M. (2011). Notch signaling activated by replication stress-induced expression of midkine drives epithelial-mesenchymal transition and chemoresistance in pancreatic cancer. Cancer Res..

[54] Lieber M., Mazzetta J., Nelson-Rees W., Kaplan M., Todaro G. (1975). Establishment of a continuous tumor-cell line (panc-1) from a human carcinoma of the exocrine pancreas. Int. J. Cancer.

[55] Tan M.H., Nowak N.J., Loor R., Ochi H., Sandberg A.A., Lopez C., Pickren J.W., Berjian R., Douglass H.O., Chu T.M. (1986). Characterization of a new primary human pancreatic tumor line. Cancer Investig..

[56] Kalinina T., Güngör C., Thieltges S., Moller-Krull M., Penas E.M., Wicklein D., Streichert T., Schumacher U., Kalinin V., Simon R. (2010). Establishment and characterization of a new human pancreatic adenocarcinoma cell line with high metastatic potential to the lung. BMC Cancer.

[57] Dancau A.M., Simon R., Mirlacher M., Sauter G. (2016). Tissue Microarrays. Methods Mol. Biol..

[58] Prianichnikov N., Koch H., Koch S., Lubeck M., Heilig R., Brehmer S., Fischer R., Cox J. (2020). MaxQuant Software for Ion Mobility Enhanced Shotgun Proteomics. Mol. Cell. Proteom..

[59] Love M.I., Huber W., Anders S. (2014). Moderated estimation of fold change and dispersion for RNA-seq data with DESeq2. Genome Biol..

[60] Ashburner M., Ball C.A., Blake J.A., Botstein D., Butler H., Cherry J.M., Davis A.P., Dolinski K., Dwight S.S., Eppig J.T. (2000). Gene ontology: Tool for the unification of biology. The Gene Ontology Consortium. Nat. Genet..

[61] Kanehisa M., Goto S. (2000). KEGG: Kyoto encyclopedia of genes and genomes. Nucleic Acids Res..

[62] Yu G., Wang L.G., Han Y., He Q.Y. (2012). clusterProfiler: An R package for comparing biological themes among gene clusters. OMICS.

[63] Liberzon A., Subramanian A., Pinchback R., Thorvaldsdottir H., Tamayo P., Mesirov J.P. (2011). Molecular signatures database (MSigDB) 3.0. Bioinformatics.

[64] Subramanian A., Tamayo P., Mootha V.K., Mukherjee S., Ebert B.L., Gillette M.A., Paulovich A., Pomeroy S.L., Golub T.R., Lander E.S. (2005). Gene set enrichment analysis: A knowledge-based approach for interpreting genome-wide expression profiles. Proc. Natl. Acad. Sci. USA.

[65] Szklarczyk D., Gable A.L., Nastou K.C., Lyon D., Kirsch R., Pyysalo S., Doncheva N.T., Legeay M., Fang T., Bork P. (2021). The STRING database in 2021: Customizable protein-protein networks, and functional characterization of user-uploaded gene/measurement sets. Nucleic Acids Res..

[66] Shannon P., Markiel A., Ozier O., Baliga N.S., Wang J.T., Ramage D., Amin N., Schwikowski B., Ideker T. (2003). Cytoscape: A software environment for integrated models of biomolecular interaction networks. Genome Res..

