# Supplementary material for: Kynureninase Is a Prognostic Biomarker of Poor Survival and Gemcitabine Resistance in Pancreatic Ductal Adenocarcinoma
Source: Int J Mol Sci. 2026 Nov 1;27(21):9820. doi: 10.3390/ijms27219820 (PMC13764116; doi:10.3390/ijms27219820)
Supplement: Supplementary file 1 [file ijms-27-09820-s001.zip › Supplementary Material.pdf]

Supplementary Figure S1. Subcellular fractionation reveals compartment-specific KYNU expression in pancreatic cell lines.

Protein lysates from L3.6pl and gemcitabine-resistant L3.6pl RES cells were subjected to subcellular fractionation, followed by Western blot analysis of KYNU expression. Detection of Cytoplasmic (GAPDH) and nuclear marker (PCNA) showed the isolation of clean fractions.

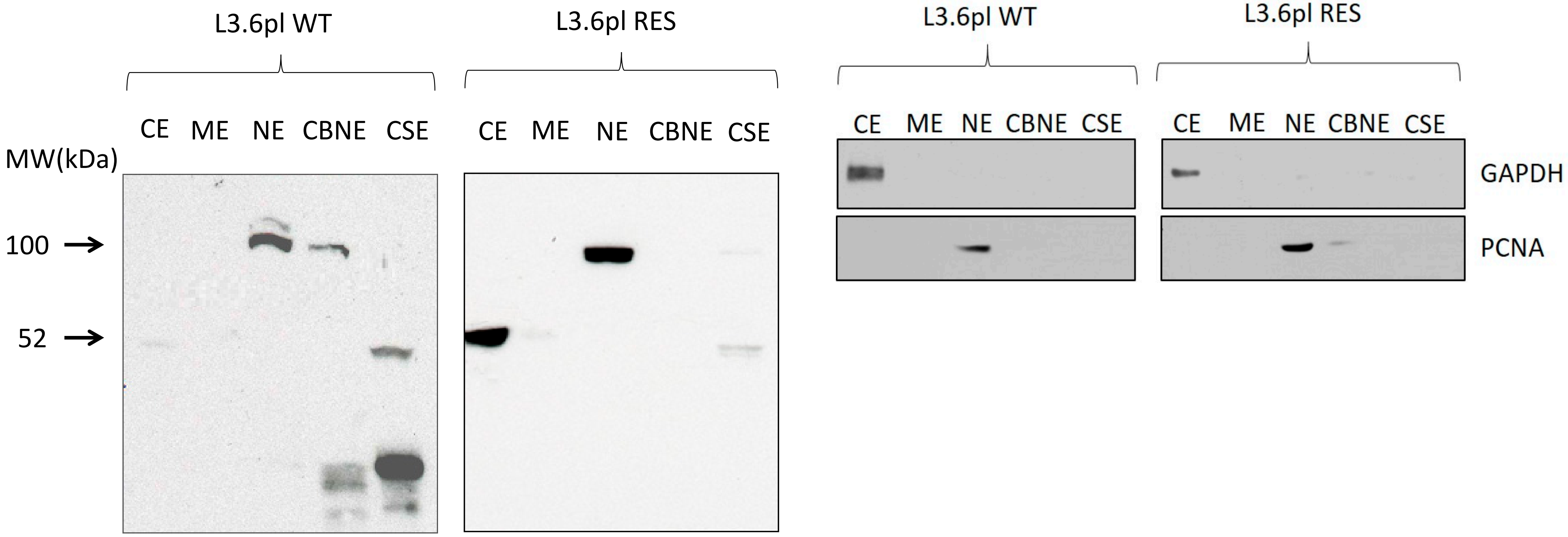

Fraction abbreviations: CE, cytoplasmic extract; ME, membrane extract; NE, nuclear extract; CBNE, chromatin-bound nuclear extract; CSE, cytoskeleton-associated extract.

Supplementary Figure S2. IFN-γ induces dose-dependent KYNU secretion in L3.6pl and L3.6pl RES cells.

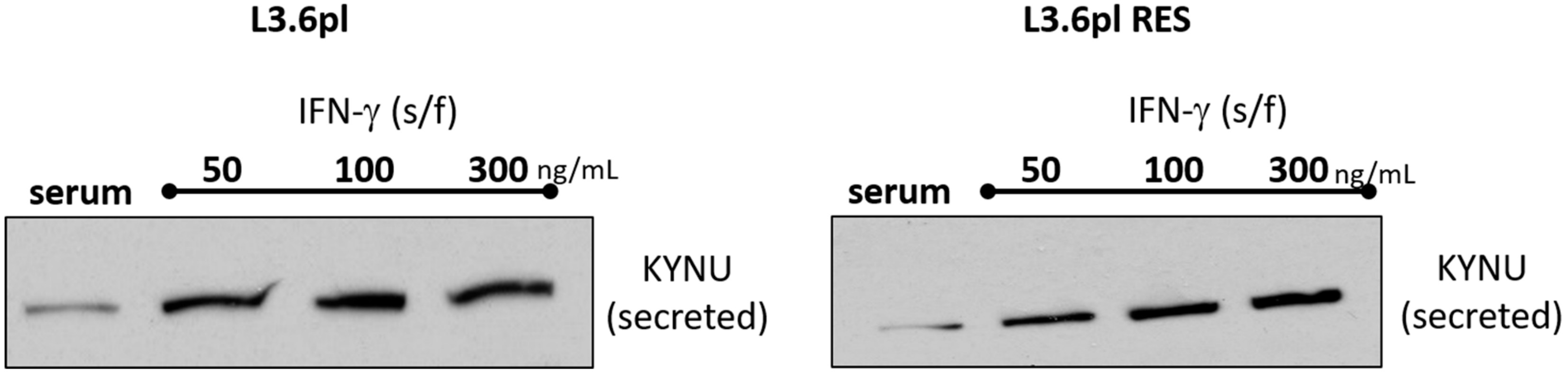

Supplementary Figure S3. Kaplan-Meier analysis of overall survival in PDAC patients stratified by KYNU expression intensity weak, moderate and strong.

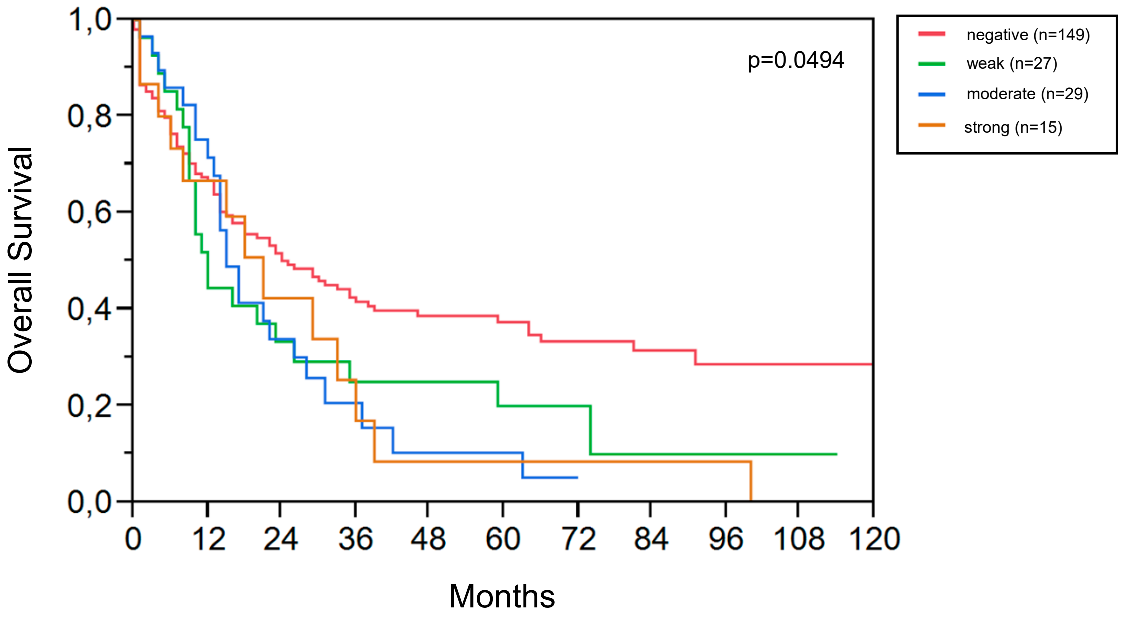

Supplementary Table S1. Association between KYNU immunostaining results and pancreatic cancer phenotype

		KYNU			
**Parameter**	**n evaluable**	**negative (%)**	**low (%)**	**high (%)**	**p value**
**All cancers**	368	63.0	13.9	23.1	
**Tumor stage**					
pT1	11	100.0	0.0	0.0	
pT2	61	55.7	23.0	21.3	**0.0217**
pT3	127	59.1	13.4	27.6	
pT4	12	66.7	16.7	16.7	
**Nodal stage**					
pN0	76	57.9	21.1	21.1	0.318
pN1	129	61.2	13.2	25.6	
**Grade**					
G1	10	80.0	10.0	10.0	
G2	115	59.1	15.7	25.2	0.7097
G3	88	58.0	17.0	25.0	

Supplementary Table S2. Available-case univariable Cox analyses of overall survival

Variable	Level	n	Events	HR (95% CI)	Coefficient *p* value	Overall *p* value
KYNU	Negative	149	90	Reference		0.027
	Low	27	22	1.51 (0.95-2.41)	0.083	
	High	44	37	1.63 (1.11-2.40)	0.013	
G stage	G1	9	5	Reference		<0.001
	G2	109	78	1.17 (0.47-2.91)	0.731	
	G3	101	88	2.31 (0.94-5.70)	0.069	
pT stage	pT1	11	5	Reference		0.004
	pT2	61	51	2.82 (1.12-7.07)	0.027	
	pT3	136	105	3.22 (1.31-7.95)	0.011	
	pT4	9	8	6.87 (2.21-21.39)	<0.001	
pN stage	pN0	86	63	Reference		0.009
	pN1	125	102	1.52 (1.10-2.08)	0.010	
Age	Per 1-year increase	284	195	1.02 (1.00-1.03)	0.006	0.005

Supplementary Table S3. Complete-case multivariable Cox analysis of overall survival

Variable	Level	n	Events	Adjusted HR (95% CI)	Coefficient *p* value	Overall *p* value
KYNU	Negative	89	64	Reference		0.697
	Low	25	22	1.13 (0.68-1.87)	0.638	
	High	41	34	0.89 (0.58-1.37)	0.599	
G stage	G1	5	2	Reference		0.001
	G2	79	56	2.09 (0.48-9.13)	0.326	
	G3	71	62	3.95 (0.91-17.22)	0.067	
pT stage	pT1	8	3	Reference		0.030
	pT2	43	37	3.37 (1.01-11.22)	0.048	
	pT3	96	73	2.99 (0.91-9.85)	0.072	
	pT4	8	7	7.24 (1.75-30.00)	0.006	
pN stage	pN0	60	41	Reference		0.001
	pN1	95	79	1.96 (1.29-3.00)	0.002	
Age	Per 1-year increase	155	120	1.01 (0.99-1.03)	0.245	0.242

All five prespecified variables forced into the model; n = 155; deaths = 120
